# Geometry-Conditioned Pair Representation and Covariance Reconstruction for Multi-Source Direction-of-Arrival Estimation with Microphone Arrays

**DOI:** 10.3390/s26217004

**Published:** 2026-11-05

**Authors:** Zixuan Liu, Wei Xiong

**Affiliations:** 1 Detroit Green Technology Institute, Hubei University of Technology, Wuhan 430068, China; 2411621306@hbut.edu.cn; 2 School of Electrical and Electronic Engineering, Hubei University of Technology, Wuhan 430068, China; 3 Department of Computer Science and Engineering, University of South Carolina, Columbia, SC 29201, USA

**Keywords:** direction-of-arrival estimation, microphone arrays, sound source localization, covariance reconstruction, spatial spectrum, source-count estimation

## Abstract

Microphone-array direction-of-arrival (DOA) estimators must cope with changes in array layout, sensor availability, and the number of active sources. We present GCPR-DOA, a geometry-conditioned pair representation that encodes each cross-spectral pair together with its microphone baseline and frequency before masked aggregation, so that one network processes arrays with 4 to 32 microphones. Two decoders use this representation: a set decoder supervised through analytic positive-semidefinite covariance reconstruction, and a spectral-moment decoder that forms a geometry-weighted steered coherence spectrum and counts sources from energy-aware features. On the LOCATA corpus, paired three-seed ablations show that geometry conditioning lowers the optimal subpattern assignment (OSPA) error by 1.35 degrees and the fixed-output angular error by 9.7 degrees. Trained on the same queries as an adapted SRP-DNN and evaluated on 46,457 common queries, the spectral-moment decoder reaches median single-source errors of 5.5–5.8 degrees, compared with 8.8–9.0 degrees, and higher active-query F1 (0.51–0.54 versus 0.47–0.49); on the three arrays other than DICIT, the two methods are comparable (5.7–5.8 versus 6.3 degrees). On the original 33,497-frame evaluation, localized F1 at ten degrees rises from 0.137 to 0.568–0.597, above all classical references.

## 1. Introduction

Microphone-array direction-of-arrival (DOA) estimation supports robot audition, spatial audio, acoustic scene analysis, and sound-source monitoring [1,2,3,4,5]. Compact arrays of microelectromechanical systems (MEMS) microphones and distributed sensors encounter changes in layout, sensor availability, and active source count. An estimator must interpret acoustic observations using the geometry that produced them and return the corresponding source directions despite noise and reverberation.

Classical approaches, including generalized cross-correlation (GCC), time-difference-of-arrival (TDOA) estimation, multiple signal classification (MUSIC), steered-response search, and sparse reconstruction, use explicit spatial models [6,7,8,9,10]. Deep neural network (DNN) estimators learn multichannel features for regression, classification, and sound-event localization [11,12,13,14,15]. Recent methods also handle multiple sources: Neural-SRP learns a steered-response representation for tracking, and SRP-DNN detects an unknown number of sources from learned direct-path phase differences and a spatial spectrum [16,17].

We focus on a representation that combines pair-specific geometry, channel-order invariance, and prediction of an unordered source set. Pairwise phase and magnitude depend on the microphone baseline and frequency as well as on the acoustic scene. When input features are tied to fixed channel identities, a network must learn these spatial associations from its training layouts. Conditioning each observation on its baseline before aggregation makes the relationship explicit. Set encoding and assignment-based prediction provide established tools for unordered inputs and outputs [18,19,20].

We propose a geometry-conditioned pair representation for DOA estimation (GCPR-DOA). A shared encoder combines the cross-spectral and coherence features of each microphone pair with the pair’s relative coordinates and frequency, and masked pooling aggregates the valid pairs. Two decoders use this representation. The set decoder (GCPR-Set) predicts directions and existence probabilities with three learned queries, and an analytic positive-semidefinite (PSD) covariance built from these predictions supplies auxiliary supervision. SubspaceNet learns a surrogate covariance for a subsequent subspace estimator [21]; GCPR-Set instead decodes the source set directly and uses the covariance only as a training target. The spectral-moment decoder (GCPR-SM) uses the geometry-conditioned pair encoding to weight a steered coherence spectrum, refines the strongest peak with spectral-moment features, and estimates the source count from energy-aware descriptors.

We developed GCPR-SM from a common-data comparison of GCPR-Set and an SRP-DNN adaptation. The observed source-activity and angular errors motivated energy-aware counting and spectrum-based direction estimation. Trained on the same LOCATA queries, GCPR-SM reaches median single-source errors of 5.5–5.8 degrees, compared with 8.8–9.0 degrees for the SRP-DNN adaptation, with similar errors on the three arrays other than DICIT.

The main contributions are:

- **A geometry-conditioned pair representation for variable arrays.** Each cross-spectral observation is encoded with its physical baseline and frequency before masked pooling. One encoder processes arrays with 4, 12, 15, and 32 microphones, and the pooled representation is invariant to joint reindexing of channels, coordinates, and masks.
- **Two decoders on this representation.** GCPR-Set predicts an unordered source set and is supervised through an analytic PSD covariance model. GCPR-SM forms a geometry-weighted steered coherence spectrum, corrects the leading direction with spectral-moment features, and counts sources with an energy-aware classifier.
- **Controlled evidence for geometry conditioning.** Paired three-seed ablations show that geometry lowers OSPA by 1.35 degrees, raises localized F1 at ten degrees by 0.034, and reduces the fixed-output angular error by 9.7 degrees, at a cost in exact-count accuracy.
- **A matched-data comparison with a learned baseline.** Trained on the same queries as SRP-DNN, GCPR-SM achieves lower median single-source error and detects more active sources. On the original LOCATA evaluation, it raises localized F1 at ten degrees from 0.137 to 0.568–0.597, above every classical reference, including those given the true source count.

Aperture, geometry, frequency, and signal-to-noise ratio (SNR) determine the directional information available from an array. Different source configurations can produce similar covariances. We therefore evaluate source sets with optimal subpattern assignment (OSPA), exact-count accuracy, and localized detection F1, and separate direction errors from detection errors using single-output readouts.

Section 2 reviews related work. Section 3 describes the representation, both decoders, and the evaluation protocols. Section 4 reports the common-data comparison, the original LOCATA evaluation, component ablations, synthetic stress tests, and runtime. Section 5 and Section 6 discuss the findings and conclude.

## 2. Related Work

### 2.1. Microphone-Array Localization and Acoustic Sensor Systems

Microphone-array localization relates propagation delays and inter-channel phase differences to source direction through the sensor geometry. Standard treatments describe steering vectors, time-delay estimation, beamforming, and broadband processing [3,6,7,22,23,24]. Studies of MEMS arrays, distributed microphone sensors, wireless acoustic sensor networks, and drone localization examine how aperture, sensor placement, synchronization, and environmental noise affect accuracy [5,25,26]. Reconfigurable arrays further require estimators that account for changes in microphone number, ordering, and placement.

Compact, distributed, and spherical arrays provide different baselines, apertures, and directional ambiguities. Their output requirements also differ: one application may need a single bearing, while another needs a set of source directions for mapping or tracking. These differences motivate representations that include microphone geometry and support variable source counts.

### 2.2. Model-Based DOA Estimation

TDOA and GCC methods estimate relative delays and infer source position or direction [6,22]. Beamforming and steered-response methods search candidate directions with a steering model; steered-response power with phase transform (SRP-PHAT) is widely used in reverberant scenes [7,27,28]. MUSIC and estimation of signal parameters via rotational invariance techniques (ESPRIT) exploit signal and noise subspaces and can reach high angular resolution under favorable conditions [8,9]. For wideband or coherent sources, coherent subspace processing and sparse reconstruction align frequency bins or impose structured sparsity on the spatial spectrum [10,29,30,31].

The performance of these methods depends on the array manifold, source count, noise model, and signal subspace. Source-number selection criteria add a separate model-dependent estimation step [32]. Broadband speech, reverberation, coherent reflections, low SNR, and calibration or geometry mismatch can violate the assumed signal model in measured recordings [33].

These models also define the quantities used in GCPR-DOA. Steering vectors map source directions to inter-sensor phases, cross-spectral matrices describe frequency-domain second-order structure, and source and noise powers determine the modeled covariance. GCPR-DOA uses cross-spectral features as inputs, the array covariance model for reconstruction supervision, and steering phases for its spectral decoder.

### 2.3. Learning-Based and Set-Valued Estimation

Convolutional and recurrent networks estimate DOA through learned feature extraction, regression, or classification. Convolutional recurrent models and Ambisonics-based systems extend this approach to time-varying scenes and to sound-event localization and detection [11,12,13,14,15]. Multi-task learning and multiple-signal-input strategies have also been investigated for DOA estimation [34]. Many designs tie their inputs or outputs to a channel order, an array geometry, or a number of sources, so a change in the recording configuration requires adaptation.

Learned methods have also been evaluated on the acoustic source LOCalization And TrAcking (LOCATA) challenge, whose data corpus is described in [35] and whose tasks and evaluation are summarized in [33]. Pak and Shin use DNN regression with phase-difference features for a two-microphone, single-source task [36]. Grinstein et al. present Neural-SRP, a learned steered-response-power tracker with released code and pretrained models [16]. Yang et al. propose SRP-DNN, which learns direct-path phase differences to localize multiple moving sources and supports unknown source counts [17]. Both methods aggregate pairwise information into a steered spatial response; GCPR-SM follows this spectrum-based route but conditions its pair weights on the physical baseline and frequency. Classical methods also combine detection with angular estimation. JADED-RIP (joint angle and delay estimator and detector via rotational invariance properties) jointly estimates multipath angles and delays and detects their number, using known orthogonal frequency-division multiplexing (OFDM) symbols and a uniform linear array (ULA) [37]. Its structured signal and array assumptions differ from the broadband acoustic setting considered here.

SubspaceNet learns a surrogate covariance through differentiable Root-MUSIC training and supplies it to a subspace DOA estimator [21]. GCPR-Set instead decodes directions and powers directly and then constructs an analytic PSD covariance for auxiliary supervision. The two methods, thus, differ in the role of the covariance: an input to a subspace estimator in SubspaceNet, and a reconstruction target for the decoded source set in GCPR-Set.

The meaning of a channel pair depends on the corresponding microphone positions. A network given only an ordered tensor must infer these spatial relationships from its training configurations; GCPR-DOA supplies them explicitly by encoding cross-spectral matrix (CSM) and coherence entries together with relative sensor coordinates. Permutation-invariant encoders aggregate unordered inputs without relying on their indexing [18,19]. Assignment-based set prediction matches unordered estimates to targets without prescribing a correspondence between output slots and physical sources [20,38,39], and multi-object evaluation similarly accounts for localization error, missed and false sources, and assignment ambiguity [40,41].

## 3. Materials and Methods

**Figure 1:**
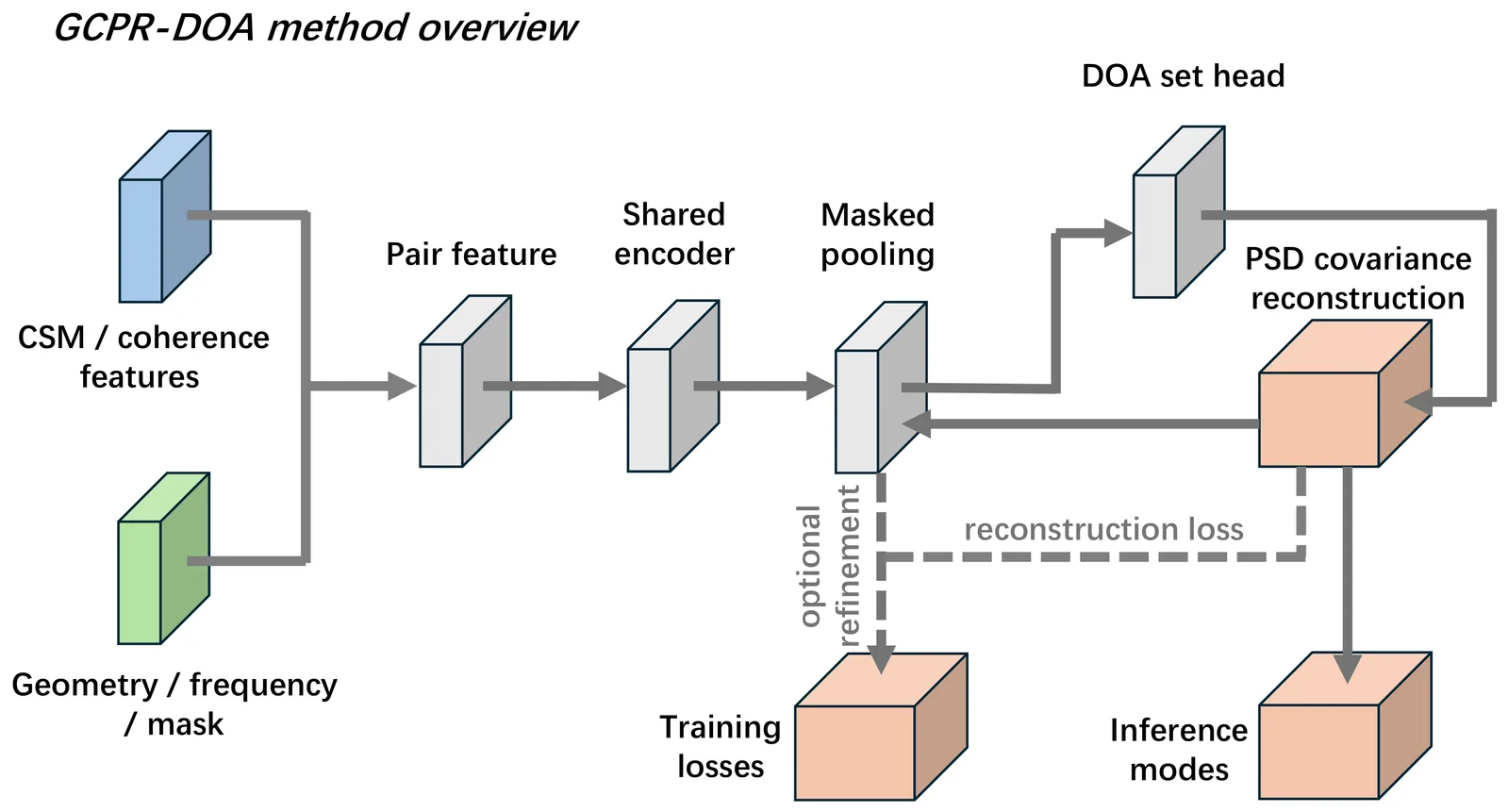
Conceptual overview of the shared representation and the set decoder (GCPR-Set). *B*, *F*, and *M* denote batch size, frequency bins, and microphones; Q=3 is the source-slot count. The complex CSM input has shape B×F×M×M. Each pair has ten channels: real, imaginary, and magnitude values for globally normalized CSM and complex coherence, three frequency-scaled baseline coordinates, and one standardized log-frequency. A shared multilayer perceptron (MLP) uses 10→128→128 channels and Gaussian error linear unit (GELU) activations. The valid-pair mask excludes invalid sensors and diagonal pairs. Masked mean pooling and frequency encoding provide context for three learned source queries; heads predict directions, existence logits, and source and noise powers. Training combines angular assignment, binary cross-entropy (BCE), and covariance consistency. The covariance return arrow represents backpropagation through the reconstruction loss. Optional refinement acts on decoded directions at inference; the primary evaluation uses zero refinement. Figure 2, Figure 3 and Figure 4 show actual masks, features, and reconstruction residuals.

**Figure 2:**
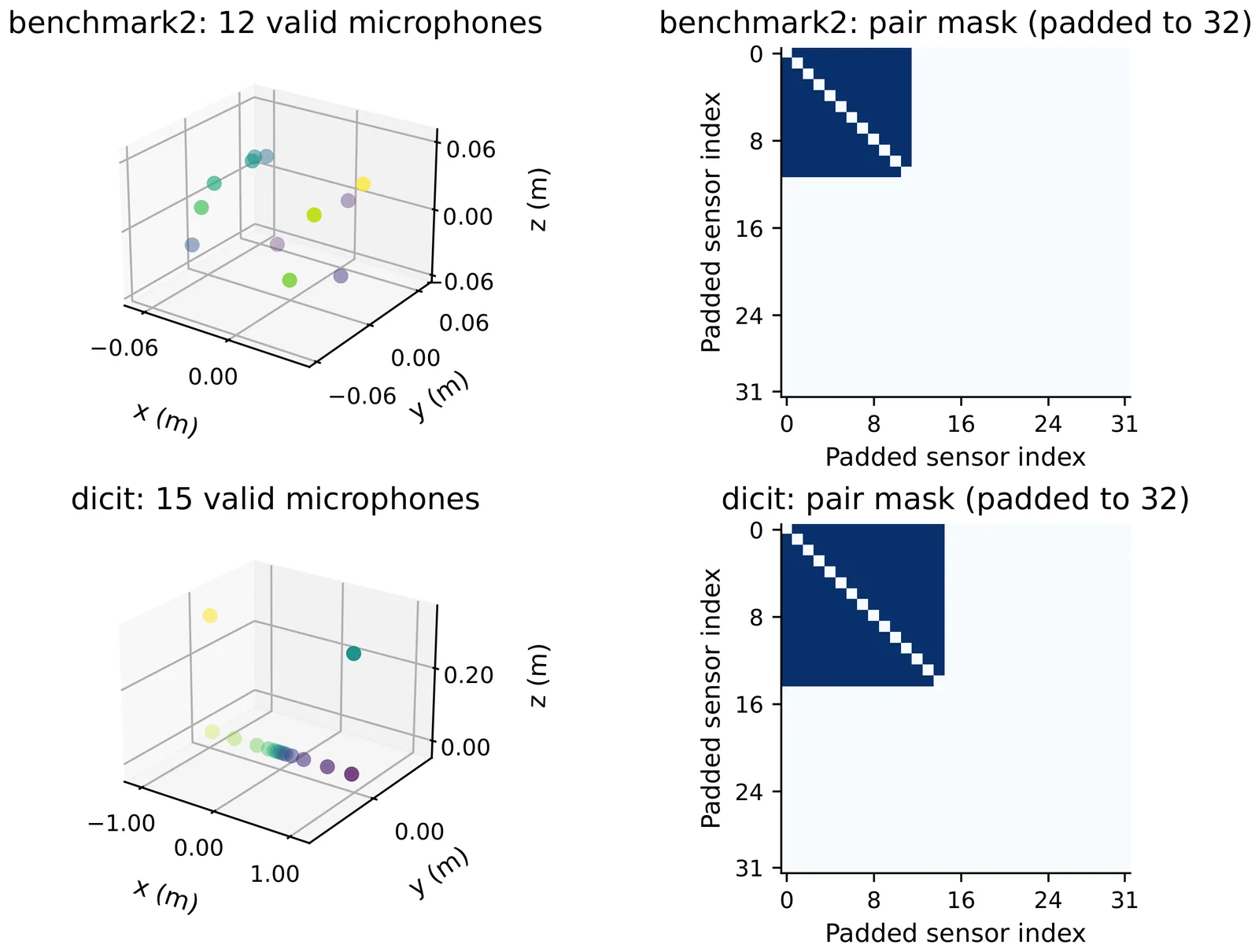

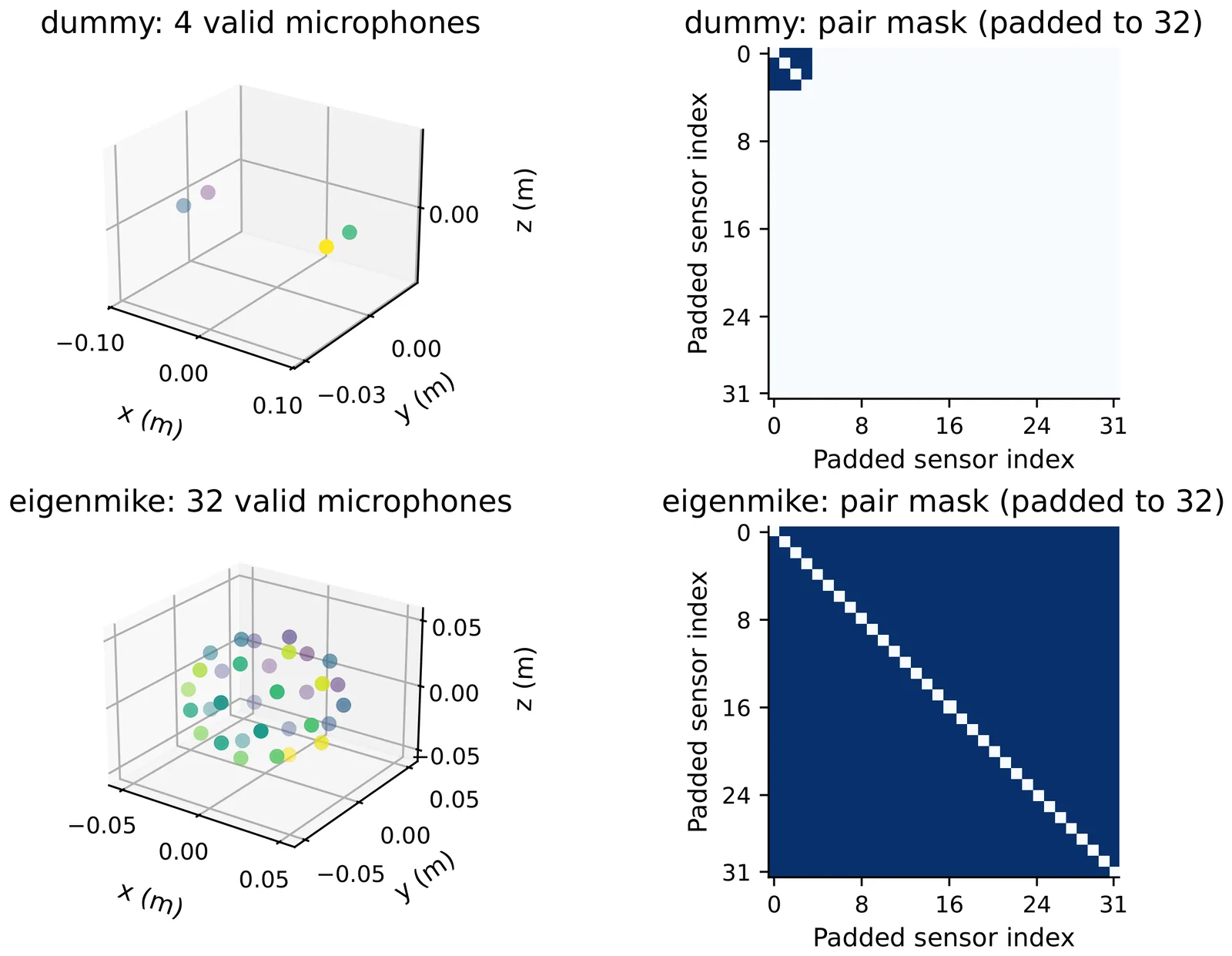
Microphone coordinates in the array coordinate frame (**left**, meters) and Boolean pair masks (**right**) from LOCATA recordings for benchmark2, dicit, dummy, and eigenmike, with 12, 15, 4, and 32 valid microphones. Sensor colors indicate channel indices. Dark mask cells denote valid off-diagonal pairs; white cells denote excluded or padded pairs. Each mask is padded to 32×32; a valid pair requires two valid microphones and m≠n. The data are from the first eligible frame of each array in the evaluation sample. DICIT denotes Distant talking Interfaces for Control of Interactive TV.

**Figure 3:**
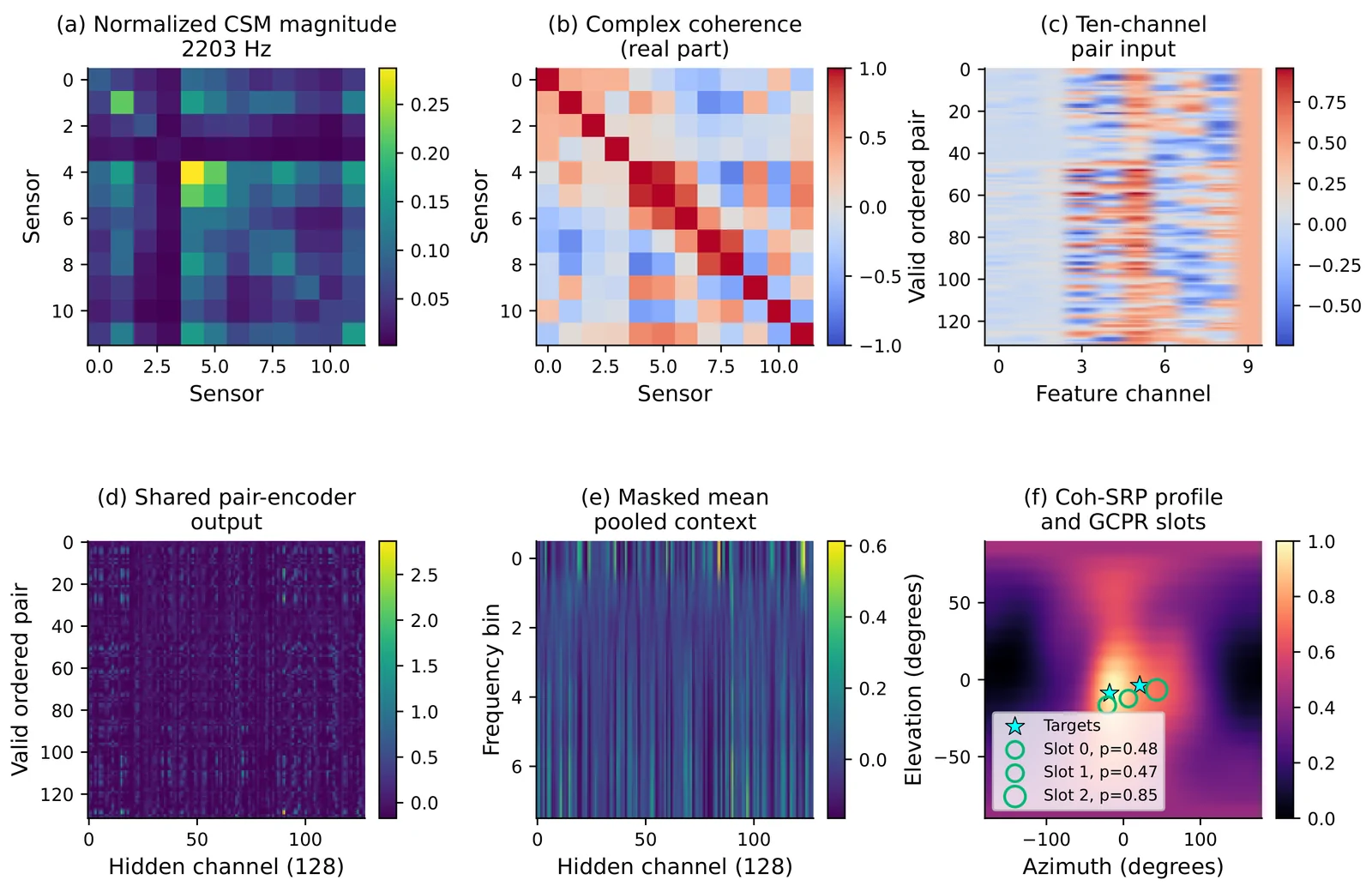
Measured features and source-slot predictions of GCPR-Set for one frame with two active talkers. The frame comes from LOCATA evaluation Task 4 (multiple moving talkers, static arrays; Table 1), recording 1, captured by the 12-microphone robot-head array (benchmark2), and has index 356 within that array-recording; it is the first eligible benchmark2 frame of Task 4 with two active talkers. Predictions come from the fully trained model with random-initialization seed 20260807, with zero refinement. (**a**) Normalized CSM magnitude $|z_{f,mn}|$ at 2203 Hz. (**b**) Real part of the complex coherence $\gamma_{f,mn}$ at 2203 Hz. (**c**) Ten-channel pair input $x_{f,mn}$ (defined in Section 3.2) at 2203 Hz for the 132 valid ordered pairs. (**d**) Shared pair-encoder output (128 channels) for the same 132 pairs at 2203 Hz. (**e**) Masked mean-pooled context, 128 channels for each of the eight frequency bins, before the frequency feature is appended. (**f**) Eight-bin coherence-weighted steered-response power (Coh-SRP) profile scaled to [0,1]; cyan stars mark the reference directions and open green circles mark the three GCPR slots with their existence probabilities *p*. All three slot probabilities exceed the threshold 0.45, so the model overestimates the source count in this frame. Tensor sizes are 1×8×12×12×10 for pair inputs, 1×8×12×12×128 for pair encodings, 1×8×129 after pooling and frequency concatenation, and 1×3×128 for queries. This example uses central processing unit (CPU) inference; aggregate scores use graphics processing unit (GPU) inference.

**Figure 4:**
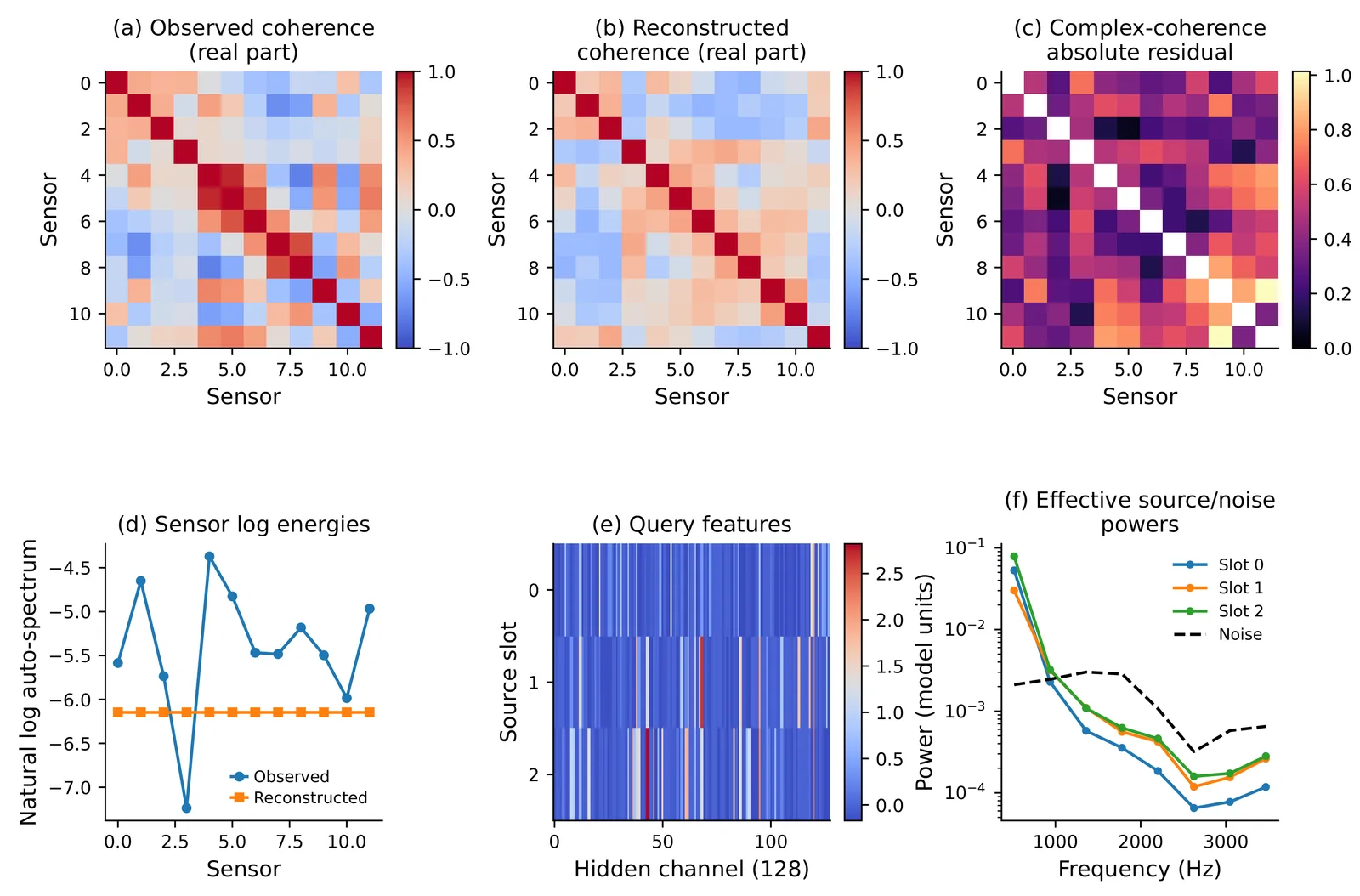
Decoder features and covariance reconstruction for the frame and model in Figure 3. (**a**) Real part of the observed complex coherence at 2203 Hz. (**b**) Real part of the reconstructed coherence at 2203 Hz, on the same color scale as (**a**). (**c**) Absolute complex-coherence residual at 2203 Hz, with excluded diagonal entries in white. (**d**) Natural-log auto-spectral energies at 2203 Hz, observed and reconstructed; the reconstructed curve is constant across sensors because the steering entries have unit magnitude and the noise power is shared across sensors. (**e**) The three decoded 128-dimensional query features. (**f**) Effective source powers $s_{i}{\hat{q}}_{f,i}$ and noise power at all eight bins, in model units. All eight reconstructed matrices are PSD, with positive eigenvalues and a minimum eigenvalue of approximately $3.19\times 10^{-4}$.

Section 3.1, Section 3.2, Section 3.3 and Section 3.4 describe the shared representation and GCPR-Set (Figure 1), and Section 3.5 describes GCPR-SM. A shared encoder first combines acoustic pair features with their geometry and frequency; masked aggregation then forms a context for the valid sensors. In GCPR-Set, learned queries decode directions and existence probabilities, with each query representing an output slot rather than a fixed source identity, and the predicted sources and powers define an analytic covariance for auxiliary supervision. Figure 2 and Figure 3 show the inputs and intermediate values, and Figure 4 shows the reconstruction and its residuals.

### 3.1. Broadband Array Model and Covariance Convention

Let $p_{m}\in R^{3}$ be the position of microphone *m*, $u_{k}\in R^{3}$ a unit source direction, *f* the frequency, and c=343 m/s the speed of sound. The steering vector and covariance convention are given in Equation (1):

(1) $$a_{m}\left(u_{k},f\right)=\exp\left(-j2\pi fp_{m}^{⊤}u_{k}/c\right),\;R_{f,mn}=E\left[\bar{X_{f,m}}X_{f,n}\right].$$

Here, $a_{m}$ parameterizes the conjugated frequency-domain observation, so its phase sign follows the stated CSM convention. To align the measured frontend with this convention, we conjugated its CSM output once and checked the phase against independently specified synthetic directions and development recordings. All measured fine-tuning, classical evaluations, and network evaluations of the archived study use these aligned inputs; synthetic pretraining and the synthetic stress tests use the original covariance generator.

For independent far-field sources and spatially white noise, the covariance approximation is given in Equation (2):

(2) $$R_{f}\approx A_{f}\left(U\right)\mathrm{diag}\left(q_{f}\right)A_{f}{\left(U\right)}^{H}+\sigma_{f}^{2}I.$$

Finite-snapshot synthetic CSMs are generated from complex source signals, steering vectors, and additive noise. Training samples have a 10% probability of no active source; otherwise the count is between one and three. This second-order far-field model omits full room-acoustic propagation, and coherent sources, near-field effects, array-housing scattering, calibration errors, and reverberation depart from its assumptions.

### 3.2. Pair Features, Pooling, and Set Decoder

The pair encoder must interpret each observation using the baseline that produced it. We, therefore, attach geometry and frequency to the acoustic features before pooling and mask invalid sensors and diagonal pairs. Let $v_{m}\in \left\{0,1\right\}$ mark a valid sensor and $w_{mn}=v_{m}v_{n}1\left[m\neq n\right]$. With invalid rows and columns excluded, Equation (3) defines the normalized CSM and complex coherence:

(3) $$z_{f,mn}=\frac{R_{f,mn}}{∥R_{f}^{\mathrm{valid}}{∥}_{F}},\;\gamma_{f,mn}=\frac{R_{f,mn}}{\sqrt{R_{f,mm}R_{f,nn}}}.$$

The Frobenius denominator and each real auto-spectral power are floored at $10^{-8}$. Here γ is complex coherence, not magnitude-squared coherence. The ten-dimensional feature is given in Equation (4):

(4) $$x_{f,mn}=[ℜz,ℑz,|z|,ℜ\gamma ,ℑ\gamma ,|\gamma |,\left(p_{m}-p_{n}\right)f/c,\ell_{f}],$$

where $\ell_{f}$ is the log-frequency standardized over the input frequency bins, with standard-deviation floor $10^{-4}$. Global CSM normalization retains relative energy across the matrix, while pair-local coherence reduces sensitivity to unequal channel powers. Both describe the same observation and are statistically dependent. The magnitudes |z| and |γ| are deterministic functions of the real and imaginary channels; they give the multilayer perceptron (MLP) explicit nonlinear amplitude cues but no independent acoustic information. Removing them reduces the first-layer weight count by 256, about 0.25% of the 102,150 parameters. Section 4.4 reports the reduced-feature comparisons.

A shared 10→128→128 MLP encodes each pair. Frequency-level pooling is given in Equation (5):

(5) $$h_{f}=\frac{\sum_{m,n}w_{mn}\phi \left(x_{f,mn}\right)}{\max(1,\sum_{m,n}w_{mn})}.$$

The appended frequency feature produces 129 channels, followed by a 129→128→128 frequency encoder and frequency averaging. A global layer-normalized context is combined with three learned 128-dimensional queries and decoded into direction vectors and existence logits. Directions are normalized with numerical floor $10^{-8}$; existence probabilities are $s_{i}=\mathrm{sigmoid}\left(b_{i}\right)$. Frequency context and query features also predict source powers, while a separate head predicts noise power.

Jointly permuting microphone coordinates, CSM rows and columns, and masks permutes the terms in Equation (5) without changing their sum, so the pooled representation is invariant to joint microphone reindexing. Mean pooling can still discard spatial information, and small aperture, linear or planar ambiguities, source coherence, low SNR, and spatial aliasing limit identifiability. Geometry conditioning lets the encoder use the baseline associated with each observation before aggregation.

Three queries limit the output to $K_{\max}=3$; thresholding retains zero to three predicted directions. Four active sources, thus, cause at least one miss, even if all reported directions are correct. Increasing the capacity requires $K_{\max}\geq 4$, training scenes and targets covering the larger count, corresponding assignment and binary cross-entropy (BCE) dimensions, and renewed threshold calibration. Section 4.9 evaluates a separately trained four-query model on synthetic scenes.

### 3.3. Assignment, Reconstruction, and Stable Training

GCPR-Set combines direct supervision of source directions and existence with an auxiliary covariance objective. The assignment step handles the unordered targets; reconstruction relates the decoded source set to second-order array observations. For each example, we compute angular costs between all prediction slots and the active reference directions. Directions are normalized, and training dot products are clipped to $\left[-1+10^{-7},1-10^{-7}\right]$ before taking arccos. The cost matrix is detached, transferred to the CPU, and matched by the Hungarian algorithm in SciPy, an open-source scientific-computing library (https://scipy.org/, accessed on 29 October 2026) [38,39]. Matching minimizes the total angular cost over injective assignments; existence probabilities do not enter that cost. Matched slots receive existence target $y_{i}=1$ and unmatched slots receive $y_{i}=0$, so with one active target and three slots, one slot receives target 1 and two receive target 0, irrespective of their predicted existence probabilities.

The direction loss is the mean matched angular distance in radians over all matched pairs in the batch. BCE with logits is averaged over all batch items and slots. If an entire batch has no target, its direction term is zero and every existence target is zero. Assignment indices are discrete; gradients flow through the selected angular terms and through BCE, not through the matching operation.

The predicted covariance is given in Equation (6):

(6) $${\hat{R}}_{f}=A_{f}\left(\hat{U}\right)\mathrm{diag}\left(s_{1}{\hat{q}}_{f,1},\ldots ,s_{K_{\max}}{\hat{q}}_{f,K_{\max}}\right)A_{f}{\left(\hat{U}\right)}^{H}+{\hat{\sigma }}_{f}^{2}I.$$

Softplus gives nonnegative source and noise powers. Together with $s_{i}\geq 0$, this ensures $t^{H}{\hat{R}}_{f}t\geq 0$ for any vector *t*, so the covariance in Equation (6) is PSD.

The reconstruction loss in Equation (7) compares complex coherence on valid off-diagonal pairs and log auto-spectral energy on valid sensors:

(7) $$L_{\mathrm{rec}}={\mathrm{mean}}_{f,m\neq n}^{\mathrm{valid}}{\left|\gamma_{f,mn}-{\hat{\gamma }}_{f,mn}\right|}^{2}+0.1{\mathrm{mean}}_{f,m}^{\mathrm{valid}}{\left(\log R_{f,mm}-\log{\hat{R}}_{f,mm}\right)}^{2}.$$

Each mean uses its own count of valid entries and frequency bins. The complete objective is given in Equation (8):

(8) $$L=L_{\mathrm{dir}}+0.25L_{\mathrm{exist}}+\lambda_{\mathrm{rec}}\left(e\right)L_{\mathrm{rec}},\;\lambda_{\mathrm{rec}}\left(e\right)=0.01\min\left(1,\max\left(0,e\right)/5\right),$$

with zero-based synthetic epoch *e*; the coefficient 0.01 is kept during fine-tuning. The three losses differ in units and reduction: direction error is in radians, BCE averages slot decisions, and reconstruction averages covariance entries. Their influence is compared through weighted gradient norms (Section 4.5). The fixed weights give the covariance term an auxiliary role, and entry averaging and the five-epoch warm-up moderate its scale.

The final linear layers for source and noise powers have zero initial weights and bias log(exp(1)−1), giving an initial power of $1+10^{-6}$ after softplus and the positive offset. Subsequent powers have no upper clamp. Training clips the global gradient norm at 5 and rejects non-finite norms; all twenty models of the archived study completed training with finite losses.

### 3.4. Inference Threshold and Optional Refinement

Unknown-count evaluations of the archived GCPR-Set retain slots with $s_{i}\geq 0.45$ and use zero refinement. The threshold was chosen on development data before the covariance correction and kept fixed for all subsequent comparisons. A later check on 17,523 corrected development-validation frames for seed 20260807 found the minimum OSPA at 0.45 on the grid 0.10:0.05:0.90, with an error of 18.30 degrees at the 30-degree cutoff; this check covers one and two active sources.

Optional refinement holds existence probabilities, source powers, and noise powers fixed and jointly updates all predicted direction coordinates using $L_{\mathrm{rec}}$. A trial update is given in Equation (9):

(9) $${\hat{u}}_{i}^{\mathrm{trial}}=\mathrm{normalize}\left({\hat{u}}_{i}-\eta \nabla_{{\hat{u}}_{i}}L_{\mathrm{rec}}\right).$$

A candidate is accepted when the reconstruction loss does not increase; otherwise the step is halved, and if no candidate is accepted, the directions are left unchanged. The default initial step is 0.05, with at most six candidate steps: $\eta =0.05\;2^{-h}$ for h=0,…,5. A budget of zero disables the update. The synthetic optimizer comparison uses a batch-averaged loss and acceptance at batch size eight; the measured implementation also supports per-frame acceptance. Refinement is disabled for all measured scores. Section 4.10 reports the sensitivity to the step and trial budget and compares three gradient updates with up to twenty iterations of joint projected Polak–Ribière-plus conjugate gradient (CG), which searches all slot direction coordinates, restarts on a non-descent direction, and normalizes trial vectors.

### 3.5. Spectral-Moment Decoder

GCPR-SM keeps the geometry-conditioned pair encoding but replaces direct direction regression with a spatial spectrum and adds energy information for source counting. Both its direction and count branches use eight-bin CSMs computed from a query-centered 512-ms segment of a 2-s observation (Section 3.7).

*Geometry-weighted steered spectrum.* A shared pair encoder combines normalized CSM, coherence, frequency, and relative microphone positions, as in Section 3.2. Averaging the encodings of each pair and its reverse makes the representation independent of pair orientation. The encoder outputs a bounded positive weight $w_{f,mn}$ for every pair and frequency, which defines the geometry-steered coherence response in Equation (10):

(10) $$P\left(u\right)=\frac{\sum_{f,m<n}w_{f,mn}\;ℜ\left\{\gamma_{f,mn}\;e^{-j2\pi f{\left(p_{m}-p_{n}\right)}^{⊤}u/c}\right\}}{\sum_{f,m<n}w_{f,mn}},\;w_{f,mn}=1+0.5\tanh h_{f,mn},$$

where $h_{f,mn}$ is the encoder output and the steering sign follows the CSM convention of Equation (11). With all weights equal to one, P(u) is the coherence-weighted steered response used by the Coh-SRP baseline; the learned weights let the network emphasize reliable pairs and frequencies while the steering phase keeps the spectrum tied to the array geometry. Three slot-specific distributions combine this response with learned ranking and frequency-resolved residuals, and their maxima on a five-degree azimuth–elevation grid, including the poles, provide candidate directions.

*Moment-based refinement.* The highest-ranked slot receives an additional correction. Microphone pairs are divided into three groups by baseline-length tertiles, and for each group and frequency the descriptor records the mean, variance, and quadrature energy of the steered coherence. With sensor-power projections, geometry statistics, and energy features, this forms a 112-dimensional descriptor, which a residual network combines with the parent score and its alignment to the peak. If the corrected peak stays at the parent grid location, a learned tangent update bounded by five degrees refines it; otherwise, a fixed 25-point tangent neighborhood with coordinate offsets of −2.5, −1.25, 0, 1.25, and 2.5 degrees is searched around the corrected peak. The other two slots keep their grid estimates. This refinement uses learned spectral features rather than the covariance loss of Section 3.4.

*Energy-aware counting.* Eighteen energy statistics retain the absolute level of the observation and of the query neighborhood; their standardization is fitted on training data. A separate count branch uses the frozen slot-spectrum model and a 94-dimensional descriptor of pooled context, energy statistics, slot existence, directional entropy, peak gaps, and inter-slot similarities. A four-class head predicts p(K=k∣x) for k=0,…,3, and the *i*-th ranked direction is retained when $\sum_{k=i}^{3}p\left(K=k|x\right)\geq 0.85$. This threshold maximizes the development active-query F1 subject to a silent false-alarm rate of at most 6%.

*Staged training.* Training proceeds from a shared weighted spectrum to slot-specific heads, frequency and moment residuals, local angular refinement, and finally the count head, with each stage keeping the parameters of the earlier stages frozen. On development data, we selected the slot-spectrum model at epoch 15, the frequency residual at epoch 4 for seed 2026101402 and epoch 2 for seed 2026101404, the moment and local refiners at epoch 8, and the count head at epoch 20. The moment loss combines directional supervision with a term that preserves already accurate training predictions; the local refiner uses array-balanced Huber supervision with bounded angular targets; and the count head uses class-weighted cross-entropy. GCPR-SM has 62,805 trainable parameters per seed, including the count branch.

### 3.6. LOCATA Data and Archived Evaluation Protocol

**Figure 5:**
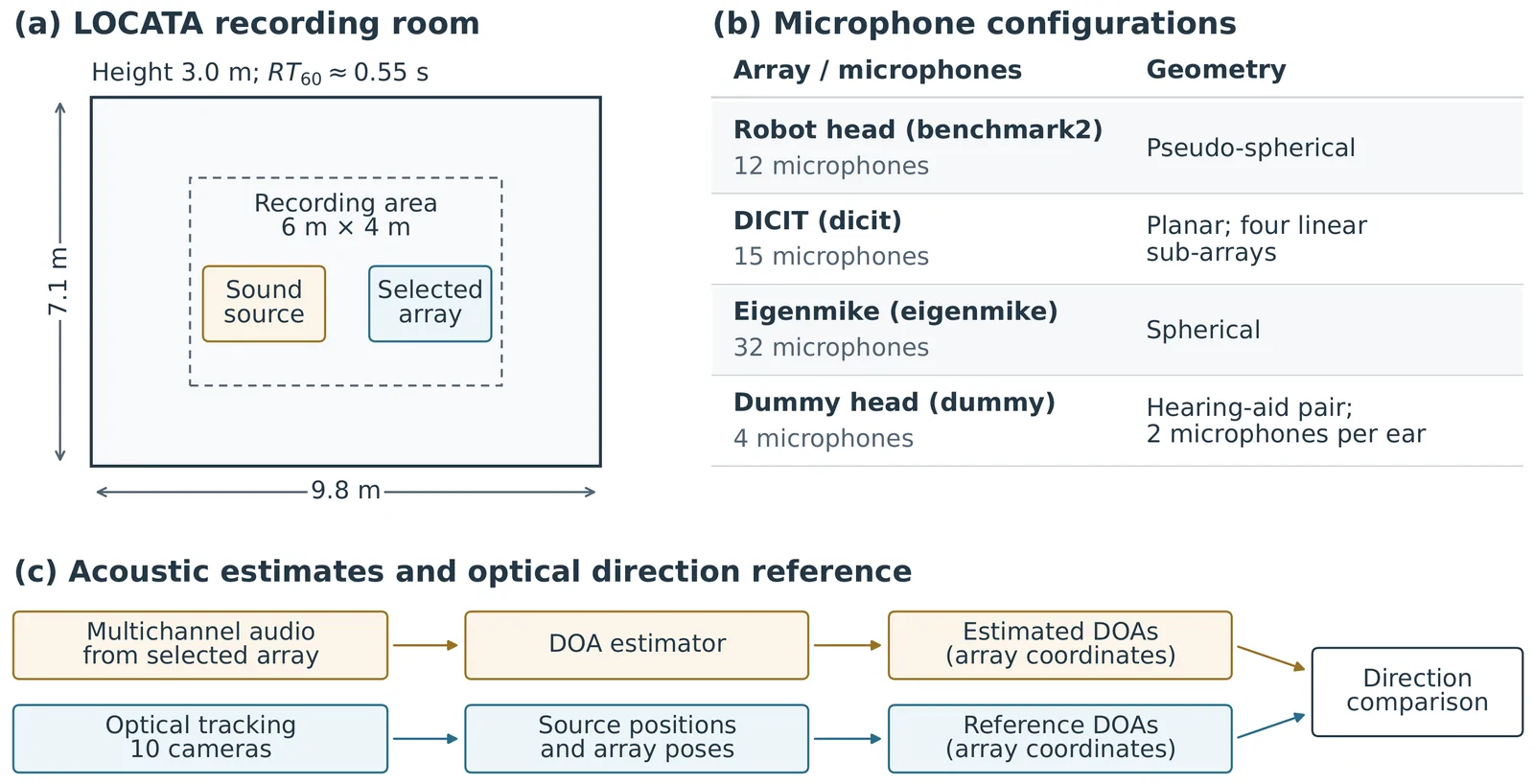
LOCATA recording environment, array configurations, and reference-direction computation [33]. (**a**) Schematic of the documented 9.8×7.1×3.0-m room and 6×4-m recording area, with $RT_{60}\approx 0.55$ s, the reverberation time for a 60-dB energy decay. Source and array blocks indicate schematic placement. (**b**) Array types and microphone counts; sensor coordinates and masks are shown in Figure 2. (**c**) Multichannel audio supplies the inputs for direction estimation. Ten optical tracking cameras supply source positions and array poses, from which array-relative reference directions are computed. Arrows show the data flow into direction comparison.

LOCATA provides measured multichannel speech together with source and array poses [33,35]; Figure 5 illustrates the recording setup. The corpus defines six tasks (Table 1), each with several recordings, and each recording is captured by up to four arrays. We use the corpus identifiers benchmark2 (12-microphone pseudo-spherical robot head), dicit (15-microphone planar DICIT array), dummy (two hearing aids with two microphones each on a dummy head), and eigenmike (32-microphone spherical Eigenmike), and call one recording captured by one array an array-recording. A task defines the recording scenario, whereas the number of sources active in a frame follows the corpus voice-activity annotations, so a frame from a multiple-source task can contain one active source.

The archived study uses 0.5-s windows and eight frequency bins between 500 and 3500 Hz. GCPR-Set variants and covariance-domain classical methods use the same CSMs and coordinate convention, and the GCPR-Set variants also share sensor masks, active-source labels, and evaluation frames. Released DNNs keep their native signal inputs and timing, with predictions aligned to common labeled frames for each comparison. Spatial aliasing depends on the microphone baselines, so the chosen band is not alias-free for every layout; Section 4.8 evaluates wider and out-of-band inputs.

The five archived full models use seeds 20260807–20260811. A seed is the integer identifier of the random initialization of one training run; for example, seed 20260807 denotes the first of the five archived full models. Seeds do not index training epochs, checkpoints, or processing stages. Each model is pretrained for thirty synthetic epochs of 500 steps at batch size sixteen and then fine-tuned for five LOCATA epochs. Core and feature ablations use seeds 20260807–20260809 with the same schedule. AdamW uses learning rate $3\times 10^{-4}$ and weight decay $10^{-4}$. Fine-tuning uses development benchmark2 recordings only, with at most 512 training frames per recording; the benchmark2 recording-3 units of development Tasks 1, 3, 4, 5, and 6 are held out for validation and model selection. The other arrays are not used for fine-tuning.

The archived evaluation uses 33,497 time-stratified sampled frames from all 132 eligible array-recordings of the evaluation partition, covering benchmark2, dicit, dummy, and eigenmike in Tasks 1, 3, 4, 5, and 6. Up to 256 frames per array-recording are selected with sampling seed 20260929, and sampling-bin weights represent 378,683 eligible deduplicated frames. The reference labels contain one or two active sources. Evaluation uses 32-bit floating-point (FP32) inference, batch size 32, no augmentation, and zero refinement. Each seed is evaluated independently, and the mean and sample standard deviation (SD) summarize the five scalar scores; no prediction ensemble is used. Classical methods run once on each of the same frames.

### 3.7. Common-Data Training and Evaluation Protocol

The common-data protocol trains every learned model on the same LOCATA queries so that differences between methods are not confounded with training data (Table 2 and Table 3). The training and development manifests contain 21,383 and 6144 queries from 19 and 10 recordings of the official development partition. The training set has 5170 queries each from benchmark2, dicit, and eigenmike and 5873 from dummy, so all four layouts occur in training. The evaluation manifest contains 46,457 queries from 82 recordings of the official evaluation partition: 35,642 active and 10,815 silent queries, with 24,239, 7852, and 3551 queries containing one, two, and three active sources. The three manifests are disjoint at the physical-recording level, where a recording is identified by its partition, task, and recording number irrespective of the array.

Each query is a 2-s waveform resampled to 16 kHz, with the labeled query time 8192 samples (0.512 s) after its start. All methods receive the same waveform interval, labels, valid sensors, and array-local coordinates, computed from double-precision pose metadata, checked for recording-wide rigidity, and stored in float32. The common-data configurations use the CSM convention in Equation (11):

(11) $$R_{f,mn}=E\left[X_{f,m}\bar{X_{f,n}}\right],\;a_{m}\left(u,f\right)=\exp\left(+j2\pi fp_{m}^{⊤}u/c\right),$$

which is the complex conjugate of Equation (1) and gives an equivalent network function once the input CSM is conjugated and the first-layer weights acting on imaginary parts change sign.

All learned models are trained from scratch with seeds 2026101402 and 2026101404, AdamW with learning rate $3\times 10^{-4}$ and weight decay $10^{-4}$, gradient-norm clipping at 1, and one parameter update per four queries; recording groups and then queries within recordings are shuffled in each epoch. No released or archived weights are loaded. The common-data GCPR-Set uses the decoder and losses of Section 3.2 and Section 3.3 with default initialization of its power heads. The SRP-DNN adaptation keeps the learned pairwise phase representation of [17]; to use the common queries, its outputs are interpolated to the query time and training minimizes the mean-squared error between the interpolated pairwise representation and the source-conditioned target. Microphone pairs are processed in micro-batches of at most sixteen, so batch-normalization statistics are computed within each micro-batch. A full-pair control removes this restriction by placing all pairs of a query in one batch-normalization group and uses FP16 computation; it was trained with seed 2026101402.

Single-stage models (GCPR-Set and both SRP-DNN variants) are trained for twenty epochs, and their checkpoint (epoch 5, 10, 15, or 20) and threshold (0.05:0.05:0.95) minimize the development group-macro OSPA, with ties broken by higher group-macro localized F1, the lower threshold, and the earlier epoch. GCPR-SM uses the staged schedule of Section 3.5 and selects its count threshold to maximize development active-query F1 subject to a silent false-alarm rate of at most 6%. To compare the methods under one rule, we also reselected the SRP-DNN thresholds with this constrained-F1 rule on development data. All models are frozen before evaluation and scored once on the evaluation manifest.

Two classical controls use the same waveforms, geometry, and eight-bin covariances on a 10,622-direction grid with nominal azimuth and elevation spacings of two and three degrees and five-degree peak separation. Covariance-domain sparse Bayesian learning (SBL) uses white-noise expectation–maximization (EM) updates with at most 2000 iterations, relative tolerance $10^{-3}$, and a normalized noise floor of $10^{-4}$, and its threshold is selected on development data; 45,134 of the 46,457 queries meet the stopping tolerance, and the others are scored as returned. A grid-count SBL control keeps twenty iterations and the counting rule of the archived study (Section 3.9).

For known-single-source errors, every method returns one direction for each of the 15,185 evaluation queries with exactly one active source in Tasks 1, 3, and 5, without a detection threshold: the highest-existence GCPR-Set slot, the top-ranked GCPR-SM direction, the first SRP-DNN directional output, or the highest classical spectrum peak. GCPR-SM is also replayed on the archived 33,497-frame sample and on the single-source subsets of Section 3.9, using its own 2-s frontend; for 4343 frames near recording boundaries, the observation is padded to its full length.

Models are fitted on the training partition; checkpoints and thresholds are selected on the development partition. The LOCATA evaluation partition had already been examined during earlier development, including the common-data GCPR-Set comparison that motivated GCPR-SM. Additional implementation and provenance details are provided in the Supplementary Material (Sections S1–S4) and the accompanying Supplementary Evidence archive.

### 3.8. Metrics and Paired Uncertainty

OSPA of order one jointly measures angular and cardinality error [40]. For predicted and true set sizes *n* and *m*, match using angular distances clipped at $d_{c}=30$ degrees and compute the score in Equation (12):

(12) $$\mathrm{OSPA}=\frac{\min_{A}\sum_{(i,j)\in A}\min\;\left(d\left({\hat{u}}_{i},u_{j}\right),d_{c}\right)+d_{c}\left|n-m\right|}{\max(n,m)}.$$

Here A ranges over rectangular matchings of min(n,m) disjoint predicted and true index pairs. When n>m, the minimizing subset is chosen from all predictions through rectangular Hungarian matching. The score is zero when both sets are empty and $d_{c}$ when only one is empty. Exact-count accuracy is the fraction of frames with n=m. Localized detection F1 at ten degrees uses angular assignment: matches within ten degrees are true positives, and unmatched predictions and targets are false positives and misses. We report means of per-frame F1, which differ from F1 computed from detections pooled across frames. OSPA combines clipped angular and count errors, whereas localized F1 rewards only accurate detections, so the two metrics can favor different estimates.

In the archived study, frame metrics are first averaged within each array-recording with sampling-bin weights; macro scores then give equal weight to the 132 array-recording means, and population-frame-weighted scores pool the same weighted frame metrics across recordings. In the common-data study, group-macro scores average 64 array–task–active-count groups, 24 of which are silent; an empty prediction for an empty target receives F1 equal to one. An always-empty predictor, thus, obtains a group-macro F1 of 24/64=0.375 and a group-macro OSPA of 30·40/64=18.75 degrees, so we also report active-query F1, the share of active queries with no detection, and the share of silent queries with a detection.

For paired contrasts, we compare matched predictions for each frame and seed and bootstrap physical recordings, keeping simultaneously recorded arrays in the same cluster, with 10,000 draws and seed 20260929. The 95% confidence intervals (CIs) resample recordings while holding the listed trained models fixed; training-seed variability is reported separately through SDs and per-seed contrasts. Synthetic intervals resample matched generated batches.

### 3.9. Classical and Released Neural Comparators

We evaluate MUSIC, its frequency-normalized form (NormMUSIC), covariance-domain SBL, and coherence-weighted steered-response power (Coh-SRP) in the archived study. All use eight-bin CSMs and a fixed 73×19 azimuth and elevation grid, with spacings of about five and ten degrees, and selected peaks are separated by at least five degrees. SBL uses twenty fixed-point iterations and noise power initialized to 5% of the mean sensor power, floored at $10^{-6}$. Coh-SRP divides cross-spectra by the geometric mean of the auto-spectral powers; this complex-coherence weighting differs from the unit-magnitude phase-transform weighting of SRP-PHAT.

We report classical methods with the true count and with an estimated count. For estimated counts, the median spectrum is subtracted and the remainder divided by peak-minus-median; the number of grid values of at least 0.5, clipped to one through three, gives the count, and a negligible dynamic range gives count zero. This rule counts above-threshold grid values rather than separated peaks and often predicts three sources. MUSIC and NormMUSIC form their unknown-count noise subspaces with $K_{\max}=3$. Peak selection then enforces the angular separation. The settings are fixed and were not tuned on evaluation data.

The released Neural-SRP models [16] and SRP-DNN [17] are evaluated on benchmark2 single-source subsets, with a known source count of one for every method. GCPR-Set returns its highest-existence slot and GCPR-SM its top-ranked direction, without selection by distance to the reference. Neural-SRP uses 253 matched active frames from 23 recordings in Tasks 1, 3, and 5, aligned by nearest timestamp within 0.06 s; SRP-DNN uses 1053 matched active frames from ten recordings in Tasks 3 and 5. Each released model is compared on its own common frames and keeps its native inputs, observation windows, and training data. These rows are external reference points; Section 4.1 compares learned models trained on common data.

## 4. Results

We first compare all learned models trained on common data (Section 4.1), then evaluate GCPR-SM and GCPR-Set on the archived LOCATA sample and on the single-source subsets of the released checkpoints (Section 4.2 and Section 4.3). Paired ablations then isolate the effect of geometry (Section 4.4), and synthetic tests examine geometry, noise, frequency, source capacity, and refinement, followed by runtime measurements.

### 4.1. Common-Data Comparison

Table 4 compares all learned models and the two classical controls on the 46,457 common evaluation queries. GCPR-SM has the lowest median single-source error, 5.52 and 5.75 degrees for its two seeds, compared with 9.02 and 8.77 degrees for the SRP-DNN adaptation and 8.63 degrees for the full-pair control. Relative to the adaptation with the same seed, the paired recording-cluster differences are −3.49 degrees (95% CI [−4.86, −2.34]) and −3.01 degrees ([−3.78, −2.24]), and relative to the full-pair control the difference is −3.11 degrees ([−4.16, −2.08]).

GCPR-SM also detects more of the active sources. Its active-query F1 at ten degrees is 0.511 and 0.541, compared with 0.468–0.485 for the adaptation under either threshold rule. The four paired F1 differences range from +0.035 to +0.064, and all of their intervals exclude zero: +0.042 [0.013, 0.072] and +0.035 [0.006, 0.066] for seed 2026101402 under the development-OSPA and constrained-F1 rules, and +0.064 [0.035, 0.094] and +0.056 [0.027, 0.087] for seed 2026101404. Against the full-pair control, the F1 difference is +0.035 [0.006, 0.063] under the development-OSPA rule and +0.027 [−0.003, 0.054] under the constrained-F1 rule. GCPR-SM returns an empty set for 14.6% and 12.3% of active queries, compared with 24.1–29.3% for the adaptation, at silent false-alarm rates of 5.7% and 6.9%, compared with 3.8–7.3%. Applying one threshold rule to all models changes the SRP-DNN scores only slightly (Table 4).

The comparison differs across arrays. On DICIT, the SRP-DNN adaptation detects almost no active sources at its selected thresholds, and its median single-source error is 114.9–122.3 degrees, compared with 5.2–5.4 degrees for GCPR-SM. Because this array dominates the four-array differences, we also compare the two methods on benchmark2, dummy, and eigenmike. On these three arrays, the median single-source errors are 5.67 and 5.80 degrees for GCPR-SM and 6.27 and 6.25 degrees for the adaptation, with paired intervals that include zero, and the silent false-alarm rates of GCPR-SM are 5.07% and 5.95%. The individual arrays favor different methods: GCPR-SM has a lower single-source error on eigenmike, with an interval that excludes zero, and a higher active F1 on dummy, whereas the adaptation has a higher active F1 on benchmark2 and eigenmike. Across benchmark2, dummy, and eigenmike, the observed median single-source errors are similar. GCPR-SM also maintains a median error near five degrees on DICIT.

The common-data GCPR-Set results motivated changes to source counting and direction estimation. At its development-selected thresholds, it returns no detection for 35,600 and 35,610 of the 35,642 active queries, and its group-macro scores (OSPA 18.76 degrees, F1 0.375) coincide with those of an always-empty predictor. Without a detection threshold, its median single-source error is 26.6–26.7 degrees. Two development-set diagnostics examine source-activity discrimination. First, its maximum slot-existence probability barely separates active from silent queries, with areas under the receiver operating characteristic curve (AUC) of 0.480 and 0.562 at the selected checkpoints and 0.461–0.562 across all candidate epochs. Second, a logistic-regression probe fitted on 896 training queries distinguishes active from silent development queries with an AUC of 0.583 when it uses 24 normalized covariance summaries, and of 0.914 when eighteen channel-energy statistics are added. The additional energy statistics improve activity discrimination in this probe. GCPR-SM uses these statistics in its count branch and estimates directions from a geometry-weighted steered spectrum.

Neither classical control approaches the learned models on both criteria: covariance-domain SBL reaches a median single-source error of 17.07 degrees but an active F1 of only 0.050, and the grid-count control reaches an active F1 of 0.231 while reporting a source in every silent query.

### 4.2. Archived LOCATA Evaluation

Table 5 reports the five archived GCPR-Set models. Macro OSPA is 23.77±0.96 degrees, population-frame-weighted OSPA is 24.00±0.81 degrees, macro exact-count accuracy is 0.507±0.156, and mean per-frame localized F1 at ten degrees is 0.137±0.011. The evaluation sample contains 30,266 one-source and 3231 two-source frames; on this predominantly single-source sample, an always-one-source rule reaches a macro count accuracy of 0.904 and a class-balanced accuracy of 0.500, compared with 0.507 and 0.416 for GCPR-Set.

GCPR-SM substantially improves on these scores (Table 6). Its two seeds reach localized F1 at ten degrees of 0.568 and 0.597, compared with 0.137 for GCPR-Set, and macro OSPA of 13.64 and 13.18 degrees, compared with 23.77 degrees. Both seeds exceed every classical method, including the true-count references, whose localized F1 ranges from 0.204 to 0.380 and whose OSPA ranges from 17.89 to 21.93 degrees. Relative to estimated-count SBL, the F1 gains are 0.297 [0.250,0.341] and 0.326 [0.283,0.368] over the 56 physical-recording clusters. Exact-count accuracy rises to 0.745 and 0.759, and GCPR-SM returns an empty set for 16.4% and 15.1% of the frames. These gains combine the new decoder, the energy-aware counting branch, the revised frontend, and training on all four layouts.

With the true count supplied, all classical methods achieve lower OSPA than GCPR-Set. With estimated counts, GCPR-Set has lower OSPA, but NormMUSIC, Coh-SRP, and SBL have higher localized F1; for example, estimated-count SBL gives an OSPA of 24.29 degrees and a localized F1 of 0.271. The SBL-minus-GCPR-Set OSPA contrast is 0.52 degrees, with cluster CI [0.003,0.989]; it combines direction errors with the errors of the grid-threshold counting rule.

**Figure 6:**
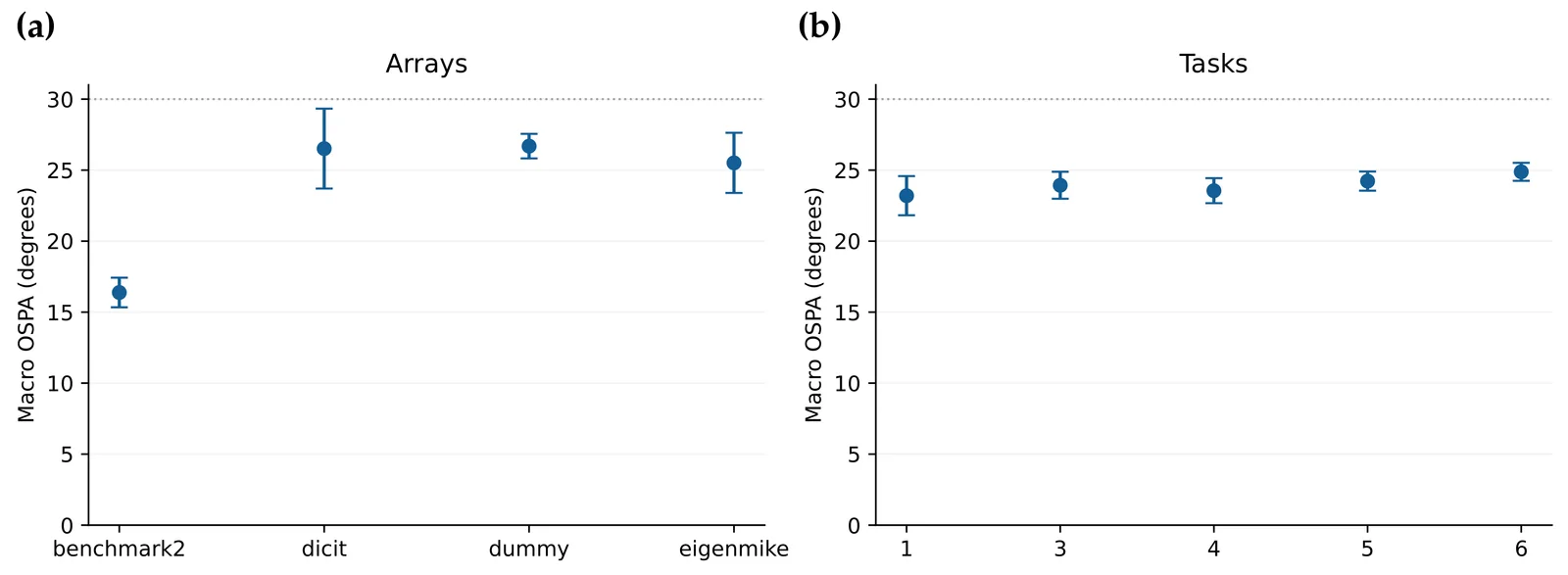
Measured OSPA of GCPR-Set (**a**) by array and (**b**) by task on the 33,497-frame sample, with equal-weight array-recording aggregation. Points show means of five independently trained models, and bars show their sample SD. OSPA uses a 30-degree cutoff, threshold 0.45, and zero refinement, as in Table 5.

Figure 6 reports GCPR-Set results by array and task. Only benchmark2 supplies its fine-tuning data. Its five-seed macro localized F1 at ten degrees is 0.392 on benchmark2 and 0.039, 0.059, and 0.058 on dicit, dummy, and eigenmike. The same network accepts all four layouts, but the archived model transfers poorly to the arrays it was not fine-tuned on; geometry, hardware response, and recording conditions change together across arrays.

### 4.3. Single-Source Comparison with Released Checkpoints

Table 7 reports the known-single-source comparisons with the released Neural-SRP and SRP-DNN checkpoints. The omnidirectional and directional Neural-SRP checkpoints achieve recording-macro OSPA of 3.52 and 4.54 degrees on their 253-frame subset, and SRP-DNN achieves 3.71 degrees on its 1053-frame subset. GCPR-SM reduces the GCPR-Set errors on these subsets from 13.17 to 5.52 degrees and from 15.00 to 7.11 and 7.03 degrees, reductions of 58% and 53%. The released checkpoints remain more accurate on these benchmark2 subsets. The released checkpoints use their original training data and protocols. In the common-data experiment (Section 4.1), GCPR-SM has lower pooled median single-source errors than the SRP-DNN adaptation across the four arrays; the largest difference occurs on DICIT.

### 4.4. Paired Effects of Geometry, Reconstruction, and Input Features

Each variant in Table 8 is paired with the full GCPR-Set at seeds 20260807–20260809, using the same inputs, training schedule, evaluation frames, threshold, and zero refinement. Removing geometry increases macro OSPA by 1.35 degrees, with conditional CI [0.66,2.08] and a positive contrast in all three seeds. Removing reconstruction increases mean OSPA by 0.78 degrees, with CI [0.44,1.13], but the seed contrasts range from −0.27 to +1.43 degrees. Population-weighted increases are 1.33 and 0.89 degrees. The no-geometry model keeps the ten-channel architecture and zeros the geometry coordinates; the no-reconstruction model changes only the reconstruction coefficient.

Geometry also improves precise localization. It raises macro localized F1 at ten degrees by 0.034 (CI [0.013,0.055]), with per-seed gains of 0.044, 0.025, and 0.034. Table 9 shows that both per-frame precision and recall at ten degrees increase, from 0.096 to 0.128 and from 0.116 to 0.159. To separate direction from count, each model also returns only its highest-existence slot on the same 30,266 single-active-source frames. Under this fixed single output, geometry lowers the mean angular error from 50.60 to 40.89 degrees, a paired difference of −9.71 degrees (CI [−14.07,−5.92]) that favors geometry in every pair, and it raises the ten-degree hit rate from 0.097 to 0.139 (difference 0.042, CI [0.019,0.064]). Geometry conditioning, therefore, improves direction estimates, not only the number of emitted sources.

Removing geometry increases macro exact-count accuracy by 7.61 percentage points, with conditional cluster CI [3.94,11.30], and removing reconstruction increases it by 4.75 points, with CI [2.13,7.44]. With geometry, the mean predicted count rises from 1.29 to 1.41 on this predominantly single-source sample; at the fixed threshold, the localization gains, thus, come with more emitted sources and lower count accuracy.

**Figure 7:**
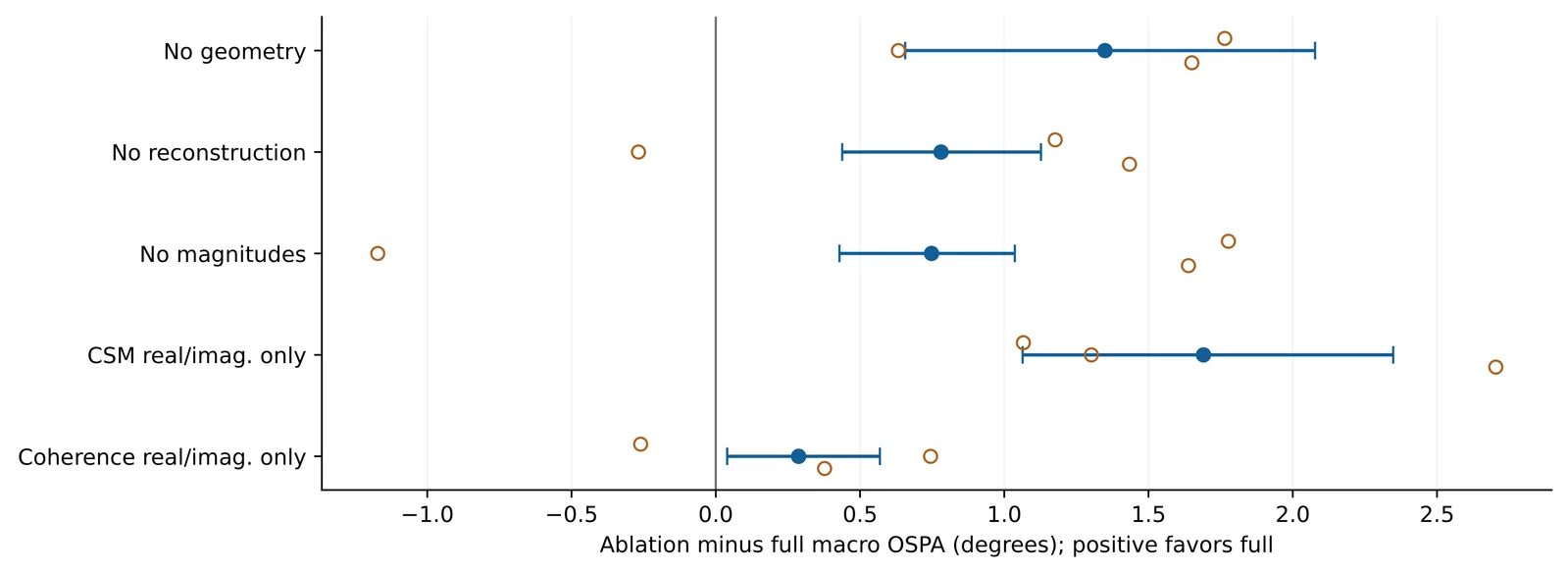
Paired measured OSPA differences for GCPR-Set, ablation minus full. Positive values favor the full model. Filled points and bars show the mean difference and 95% physical-recording-cluster bootstrap CI with the three trained models held fixed. Open markers show individual seed differences.

The feature ablations remove both explicit magnitudes, leaving eight input channels; keep only the normalized-CSM real and imaginary values, with six channels including geometry and frequency; or keep only the coherence real and imaginary values, also with six channels. Their macro OSPA increases are 0.75, 1.69, and 0.29 degrees. For magnitude removal, the three seed differences are +1.78, −1.17, and +1.64 degrees. The full feature set has the lowest mean OSPA, although its acoustic channels remain dependent; because removing channels also removes input-layer capacity, these ablations change feature content and parameter count together. Figure 7 shows the paired OSPA differences.

### 4.5. Loss-Scale and Stability Diagnostics

We computed the unweighted and weighted gradient norm of each loss in Equation (8) for 21 evaluations of development-validation batches at selected checkpoints of three full-model seeds, with batch size sixteen. Table 10 reports medians across these evaluations. Weighted all-parameter norms are 3.294 for direction, 0.710 for existence, and 0.0458 for reconstruction; on the shared encoder, the corresponding medians are 2.494, 0.666, and 0.0444. All measured gradients are finite, and the weighted reconstruction gradient is about 70 times smaller than the direction gradient, consistent with its auxiliary role.

### 4.6. Spherical Geometry and Synthetic Stress Cases

The synthetic spherical comparison evaluates the full, no-geometry, and fixed-channel-order models on the same 4000 scenes with five seeds. Sensor counts are 4, 6, 8, 10, 12, 16, 20, 24, and 32, and all models use synthetic pretraining without LOCATA fine-tuning. The five scenarios are nominal conditions, eight snapshots, 5-mm sensor-position perturbation, 25% sensor dropout, and coherent sources (Table 11).

Aggregate OSPA is 26.62 degrees for the full model, 28.92 degrees without geometry, and 27.25 degrees with a fixed channel order. The full-minus-comparator differences are −2.29 degrees, with batch-cluster CI [−2.51,−2.08], and −0.63 degrees, with CI [−0.85,−0.40]. Geometry conditioning helps in every scenario, and the full model also outperforms the fixed-order model except under position perturbation and dropout, where the intervals include zero. These tests use free-field synthetic covariances; the measured eigenmike results also include room reverberation and hardware response.

### 4.7. Noise Robustness

**Figure 8:**
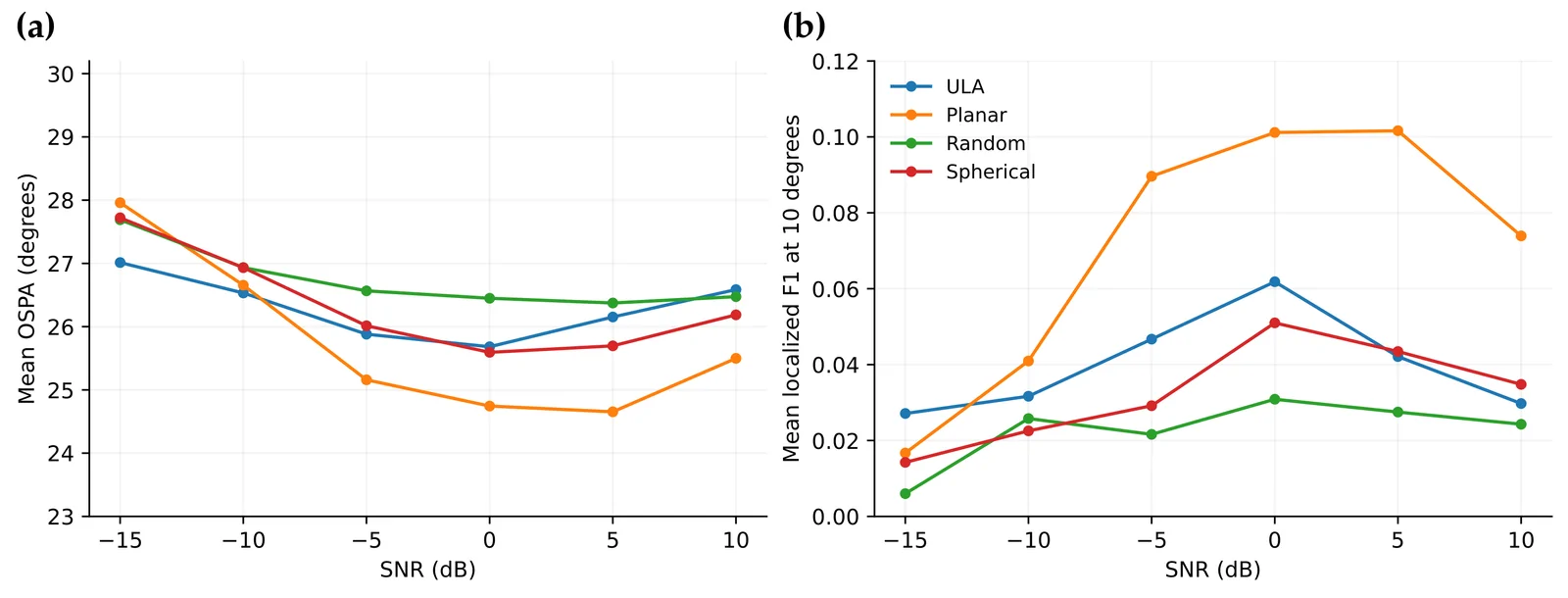
Synthetic SNR sweep for five pretrained GCPR-Set models with twelve sensors and no LOCATA fine-tuning. Each geometry/SNR condition has 800 model/scene evaluations across seeds. (**a**) Mean OSPA with a 30-degree cutoff. (**b**) Mean per-frame localized detection F1 within ten degrees. All conditions use existence threshold 0.45 and zero refinement; noise varies in the covariance-domain simulation.

Figure 8 evaluates five synthetically pretrained models with twelve microphones, four geometry families (uniform linear, planar, random, and spherical), and six SNRs from −15 to 10 dB. Each geometry and SNR condition has 800 frames across seeds, giving 19,200 model–scene evaluations, with threshold 0.45, the 30-degree cutoff, and zero refinement. Aggregate OSPA is 27.60 degrees at −15 dB and 26.19 degrees at 10 dB; their matched difference is 1.41 degrees, with batch-cluster CI [1.26,1.57], on 3200 paired frames across 400 generated-batch clusters.

OSPA varies non-monotonically with SNR for some geometries: some 0 and 5 dB conditions outperform 10 dB, and localized F1 remains low for this pretrained set decoder. These curves characterize covariance-domain noise in the synthetic signal model. Room-acoustic propagation and interference are excluded from the simulation.

### 4.8. Frequency Extrapolation and Spatial Aliasing

The frequency test uses five pretrained models on matched twelve-sensor ULAs at −5 dB, with 0.30-m aperture and spacing of about 0.0273 m, whose first half-wavelength aliasing limit is about 6.29 kHz. Each band has 800 paired frames across seeds. Table 12 reports OSPA, which clips angular errors at 30 degrees, together with the untruncated matched angular error.

Expanding the band from 500–3500 Hz to 500–5500 Hz increases OSPA by 2.57 degrees, with conditional CI [2.10,3.07], even though the wider band stays below the nominal aliasing limit; the increases for 500–7000 Hz and 3500–7000 Hz are 2.94 and 3.18 degrees. The set decoder is, thus, sensitive to frequencies outside its training band. The wider-band tests combine out-of-band inputs with potential aliasing and do not separate the two effects.

### 4.9. Source-Capacity Extension and Failure Cases

We compare a separately trained four-query synthetic set decoder with the three-query model at seed 20260807 on 1280 paired scenes, spanning zero to four true sources across four geometry families. All true targets remain in scoring, including four-source overload cases for the three-query model. Table 13 reports OSPA and predicted counts.

On four-source scenes, the four-query model reduces OSPA from 27.28 to 23.84 degrees, a paired difference of −3.44 with batch-cluster CI [−4.22,−2.54]; because OSPA includes cardinality error, this change reflects both direction and count. On empty scenes, however, the four-query model predicts 3.54 sources on average and has OSPA 30.00 degrees, compared with 1.07 sources and 13.80 degrees for the three-query model, and its single-source OSPA is also worse. A larger query head, thus, lowers four-source OSPA at the cost of false detections in silence.

### 4.10. Optional Refinement Sensitivity and Joint Optimization

Table 14 compares batch-mean refinement on 160 matched twelve-sensor spherical scenes for seed 20260807, with batch size eight. Three gradient updates with backtracking give an OSPA of 24.82 degrees, compared with 24.63 degrees for joint projected CG with at most twenty iterations. The CG-minus-backtracking difference is −0.19 degrees, with batch-cluster CI [−0.30,−0.10], and the reconstruction loss decreases by 0.00535. Mean CPU refinement time rises from 18.26 to 101.41 ms per batch, about 5.55 times longer for CG, so the small error reduction requires substantially more computation.

The step-size sweep uses 160 spherical and 160 ULA validation scenes, one pretraining seed, and three updates, with initial steps of 0.01, 0.025, 0.05, 0.10, and 0.20 and trial budgets of 0, 2, 4, and 6. Budgets 2, 4, and 6 give identical scores because the first candidate step is always accepted in this sweep. At budget six, increasing the step from 0.05 to 0.20 changes OSPA from 25.573 to 25.558 degrees on spherical scenes and from 24.609 to 24.599 degrees on ULA scenes. Neither the default step of 0.05 nor the six-trial budget is critical; the complete grid is included in the reproducibility materials.

### 4.11. Runtime Measurements

Table 15 profiles the full GCPR-Set model of seed 20260807 on one fixed frame per array, using one CPU thread and an NVIDIA GeForce RTX 4060 Laptop GPU (NVIDIA Corporation, Santa Clara, CA, USA), with Python 3.10.11 (Python Software Foundation, Beaverton, OR, USA) and PyTorch 2.7.1 (PyTorch Foundation; https://pytorch.org/, accessed on 29 October 2026) with Compute Unified Device Architecture (CUDA) 12.8. Network-only and classical spectral timings use twenty warm-ups and 100 synchronized repetitions; frontend and pipeline timings use ten warm-ups and fifty repetitions. All entries are batch-one medians. CPU network-only latency ranges from 1.32 to 7.91 ms and GPU latency from 2.39 to 2.64 ms, so GPU inference is not uniformly faster at batch size one.

Pipeline timing starts from an already available audio window and includes reading it, constructing the CSM, loading metadata, transferring data, running the network, thresholding, and returning CPU outputs, with a warm operating-system file cache. Median times are 5.13–22.22 ms on CPU and 8.02–23.15 ms on GPU. Audio acquisition for the 0.5-s window, continuous-stream scheduling, application integration, and cold-cache behavior are excluded. Classical entries cover spectrum computation, counting, and peak selection from a preloaded CSM. GCPR-SM has 62,805 trainable parameters per seed, fewer than the 102,150 of GCPR-Set and the 861,696 of the full-pair SRP-DNN control; its grid search and moment computation add inference cost that these timings do not cover.

## 5. Discussion

Geometry conditioning improves localization in the paired GCPR-Set experiments. The three-seed averages show lower OSPA and higher localized precision and recall. Geometry also reduces the fixed-output angular error in every pair, with a mean reduction of 9.7 degrees. Synthetic spherical tests favor geometry conditioning under sensor perturbation, dropout, and coherent sources. The two decoder pipelines yield different source-set accuracy: GCPR-Set has lower localized F1 on arrays outside its fine-tuning data and predicts nearly empty sets when trained from scratch on the common LOCATA queries. GCPR-SM uses geometry-conditioned pair encoding to weight a steered coherence spectrum and reaches median single-source errors of 5.5–5.8 degrees under the same training data as SRP-DNN.

Geometry enters the two decoders through different operations. GCPR-Set conditions pair features on their baselines and regresses directions from a pooled context. In GCPR-SM, the steering phase in Equation (10) evaluates each candidate direction using the array geometry, while the pair encoder learns weights for microphone pairs and frequencies. Uniform weights recover the Coh-SRP response. The learned spectral model reaches pooled median single-source errors of 5.7–5.8 degrees on benchmark2, dummy, and eigenmike and 5.2–5.4 degrees on DICIT. Energy statistics supply the count branch with activity cues, consistent with the development-set probe results.

On the four-array common-data evaluation, GCPR-SM achieves lower pooled median single-source error and higher active F1 than the SRP-DNN adaptation. The largest angular differences occur on DICIT. On the pooled benchmark2, dummy, and eigenmike queries, the two methods have similar median errors, with paired difference intervals that include zero. Results for individual arrays favor different methods. The released SRP-DNN and Neural-SRP checkpoints achieve lower errors on their benchmark2 single-source subsets using their original training data and native protocols.

Covariance reconstruction provides auxiliary supervision for GCPR-Set. It reduces mean OSPA by 0.78 degrees: OSPA decreases in two seeds and increases in the third. Its weighted gradients are about 70 times smaller than the direction gradients. The reconstruction in Figure 4 has equal diagonal powers across sensors because its steering entries have unit magnitude and its white-noise power is shared. The measured auto-spectral energies vary by channel.

The common-data comparison uses shared queries and method-specific training protocols. The SRP-DNN adaptation uses query-level supervision and pair micro-batching, and the full-pair control contributes one seed. The separate Neural-SRP comparison uses released checkpoints. All four array layouts are represented in the common training data and evaluated in the common-data comparison. The LOCATA evaluation partition had been examined during earlier development. Training budgets differ: GCPR-SM uses staged fitting and development selection, while the common-data baselines use single-stage training. The measured evaluation covers up to three simultaneous sources. The four-query synthetic pilot improves four-source OSPA and increases false detections in silence.

The next steps are native-protocol comparisons with Neural-SRP and SRP-DNN on common data, transfer tests on held-out arrays and new recordings, larger simulated and measured training corpora, and covariance-consistent peak refinement.

## 6. Conclusions

We presented GCPR-DOA, a geometry-conditioned pair representation that encodes each cross-spectral pair with its baseline and frequency so that one network can process microphone arrays of different sizes and layouts. Paired ablations show that geometry conditioning improves localization within a fixed architecture: OSPA decreases by 1.35 degrees, localized F1 at ten degrees increases by 0.034, and the fixed-output angular error decreases by 9.7 degrees, at a cost of 7.6 percentage points in exact-count accuracy.

Built on this representation, the spectral-moment decoder combines a geometry-weighted steered coherence spectrum, moment-based peak refinement, and energy-aware counting. Trained on the same LOCATA queries as an SRP-DNN adaptation, it reaches median single-source errors of 5.5–5.8 degrees, compared with 8.8–9.0 degrees, detects more active sources, and is comparable to SRP-DNN on the three arrays other than DICIT. On the original LOCATA evaluation, it raises localized F1 at ten degrees from 0.137 to 0.568–0.597, above all classical references. The reported LOCATA comparisons show higher source-detection F1 and lower directional errors for GCPR-SM than for GCPR-Set.

## Figures and Tables

**Table 1:** LOCATA tasks as defined by the corpus [33,35]. The last column marks the tasks that contribute frames to the archived evaluation.

Task	Sources	Source Motion	Arrays	Archived Evaluation
1	Single	Static loudspeaker	Static	Yes
2	Multiple	Static loudspeakers	Static	No
3	Single	Moving talker	Static	Yes
4	Multiple	Moving talkers	Static	Yes
5	Single	Moving talker	Moving	Yes
6	Multiple	Moving talkers	Moving	Yes

For Tasks 5 and 6, array positions and orientations are provided with the recordings. Task labels describe the scenario; the number of active sources in a frame follows the voice-activity annotations.

**Table 2:** Model configurations and their roles in the evaluation.

Configuration	Architecture and Training	Role
GCPR-Set (archived)	Set decoder with covariance supervision (Section 3.2, Section 3.3 and Section 3.4); synthetic pretraining and benchmark2 fine-tuning	Archived evaluation, component ablations
GCPR-Set (common data)	Same decoder and losses; trained from scratch on the common LOCATA queries	Common-data comparison
GCPR-SM	Spectral-moment decoder with energy-aware counting (Section 3.5); staged training on the common LOCATA queries	Common-data comparison, archived evaluation
SRP-DNN adaptation	Learned pairwise phase representation; query-level supervision; pair micro-batches of at most sixteen pairs	Common-data comparison
SRP-DNN full-pair control	As above, with all pairs of a query in one batch-normalization group; one seed	Common-data comparison

**Table 3:** Shared conditions of the common-data comparison and method-specific elements.

Item	Setting
Queries	21,383 training and 6144 development queries with identical identifiers for all learned models; 46,457 evaluation queries
Recordings	19 training and 10 development recordings from the official development partition; 82 evaluation recordings from the official evaluation partition; disjoint at the physical-recording level
Observation	Common 2-s, 16-kHz waveform interval; query time 0.512 s after its start; common labels, valid sensors, and array-local coordinates
Optimization	From scratch; AdamW, learning rate $3\times 10^{-4}$, weight decay $10^{-4}$, gradient-norm clipping at 1, one update per four queries; seeds 2026101402 and 2026101404
Model selection	Development data only; checkpoints at epochs 5, 10, 15, and 20 (single-stage models) and thresholds 0.05:0.05:0.95
Method-specific	Input representation, network, and loss; SRP-DNN query adaptation and pair micro-batching; GCPR-SM staged fitting and constrained-F1 threshold rule

**Table 4:** Common-data comparison on 46,457 evaluation queries (35,642 active and 10,815 silent). All learned models are trained on the same queries; epochs and thresholds are selected on development data. Single-source errors use the 15,185 queries with one active source in Tasks 1, 3, and 5.

		Macro	Active	Empty	Silent	Single	Macro
Method/Seed	Rule	F1@10	F1@10	Active (%)	Alarms (%)	Median (°)	OSPA (°)
GCPR-SM/1402	Constr. F1	0.649	0.511	14.63	5.71	5.52	11.33
GCPR-SM/1404	Constr. F1	0.655	0.541	12.26	6.88	5.75	11.22
SRP-DNN adapt./1402	Dev. OSPA	0.632	0.468	27.77	6.13	9.02	11.93
SRP-DNN adapt./1402	Constr. F1	0.633	0.475	24.10	7.26	9.02	11.86
SRP-DNN adapt./1404	Dev. OSPA	0.647	0.477	29.34	3.75	8.77	11.43
SRP-DNN adapt./1404	Constr. F1	0.646	0.485	25.96	5.68	8.77	11.44
Full-pair ctrl./1402	Dev. OSPA	0.632	0.475	28.18	6.22	8.63	12.07
Full-pair ctrl./1402	Constr. F1	0.634	0.484	27.57	7.32	8.63	12.00
GCPR-Set/1402	Dev. OSPA	0.375	0.0005	99.88	0.12	26.63	18.76
GCPR-Set/1404	Dev. OSPA	0.375	0.0002	99.91	0.06	26.73	18.76
Cov. SBL (EM)	Dev. sel.	0.372	0.050	–	7.14	17.07	18.89
Grid-count SBL	Fixed	0.135	0.231	–	100.0	13.94	26.62

Macro values average 64 array–task–source-count groups, including 24 silent groups. Active F1@10 is the mean per-query localized F1 at ten degrees over active queries. Empty active is the share of active queries with no detection; silent alarms is the share of silent queries with at least one detection. Single-source errors are untruncated medians of one direction per query, read out without a detection threshold, so they do not depend on the threshold rule. Dev. OSPA selects the lowest development group-macro OSPA; Constr. F1 maximizes development active F1 subject to a silent false-alarm rate of at most 6%. Rule is the threshold-selection rule. OSPA uses order one and a 30-degree cutoff.

**Table 5:** GCPR-Set scores on 33,497 sampled frames from 132 array-recordings, with a 30-degree OSPA cutoff. Mean and sample SD summarize five independently trained models.

Seed	Macro OSPA (°)	Weighted OSPA (°)	Count Acc.	Loc. F1@10
20260807	23.17	23.60	0.646	0.151
20260808	24.33	24.02	0.301	0.124
20260809	23.32	23.87	0.424	0.134
20260810	25.18	25.32	0.490	0.131
20260811	22.88	23.17	0.674	0.144
Mean ± SD	23.77±0.96	24.00±0.81	0.507±0.156	0.137±0.011

Count accuracy and localized F1 are macro values. Loc. F1@10 is the mean of per-frame localized-detection F1 at ten degrees. SD is the sample standard deviation across five trained models.

**Table 6:** Classical methods and GCPR-SM on the same 33,497 sampled frames. True-count rows receive the active source count; all other rows estimate it.

Method	Count	Macro OSPA	Weighted OSPA	Count Acc.	Loc. F1@10
*A. True-count localization references*					
MUSIC	oracle	21.93	23.27	1.000	0.204
NormMUSIC	oracle	18.89	20.04	1.000	0.339
Coh-SRP	oracle	18.40	19.37	1.000	0.374
SBL	oracle	17.89	19.23	1.000	0.380
*B. Estimated count with grid-threshold counting*					
MUSIC	estimated	26.52	26.55	0.021	0.147
NormMUSIC	estimated	25.45	25.27	0.000	0.229
Coh-SRP	estimated	25.07	25.19	0.000	0.248
SBL	estimated	24.29	24.52	0.054	0.271
*C. Learned models, estimated count*					
GCPR-Set (mean of five)	estimated	23.77	24.00	0.507	0.137
GCPR-SM/1402	estimated	13.64	–	0.745	0.568
GCPR-SM/1404	estimated	13.18	–	0.759	0.597

OSPA is in degrees; count accuracy and localized F1 are macro values. Coh-SRP uses complex-coherence weighting; SRP-PHAT uses unit-magnitude phase weighting. Group A receives the true count. Group B counts above-threshold grid values and often predicts three sources, so its OSPA combines direction errors with errors from that counting rule. GCPR-SM uses its own 2-s frontend on the same frames and is trained on the common-data queries (Section 3.7).

**Table 7:** Recording-macro OSPA in degrees on the matched known-single-source subsets of the released DNN checkpoints. Each method returns one direction per frame; errors are clipped at 30 degrees.

Released Reference	Frames	Records	Reference	GCPR-Set ± SD	GCPR-SM (1402/1404)
Neural-SRP (omni)	253	23	3.52	13.17±1.30	5.52/5.52
Neural-SRP (dir.)	253	23	4.54	13.17±1.30	5.52/5.52
SRP-DNN	1053	10	3.71	15.00±1.28	7.11/7.03

Neural-SRP uses Tasks 1, 3, and 5; SRP-DNN uses Tasks 3 and 5; all subsets use benchmark2. The released checkpoints keep their native inputs, observation windows, and training data. For GCPR-Set, the reference-minus-GCPR differences and recording-cluster CIs are −9.64 [−11.40, −7.98], −8.63 [−10.37, −6.99], and −11.29 [−12.16, −10.13] degrees for the three rows.

**Table 8:** Paired ablation-minus-full OSPA contrasts for GCPR-Set in degrees, with 95% cluster CIs conditional on the same three trained seeds. Positive contrasts favor the full model.

Ablation	Input Dim.	Macro Δ [CI]	Weighted Δ [CI]	Seed Range
No geometry	10	1.35 [0.66, 2.08]	1.33 [1.08, 1.57]	+0.63 to +1.76
No reconstruction	10	0.78 [0.44, 1.13]	0.89 [0.77, 1.02]	−0.27 to +1.43
No magnitudes	8	0.75 [0.43, 1.04]	0.93 [0.76, 1.08]	−1.17 to +1.78
CSM real/imag. only	6	1.69 [1.06, 2.35]	1.52 [1.21, 1.82]	+1.07 to +2.70
Coherence real/imag. only	6	0.29 [0.04, 0.57]	0.15 [0.06, 0.24]	−0.26 to +0.74

The final column gives the range of three macro seed differences. No-geometry zeros the geometry channels. Six-channel branches retain the real and imaginary parts of normalized CSM or coherence, three geometry values, and frequency; explicit magnitudes are omitted. CIs resample recording clusters with the three trained models held fixed.

**Table 9:** Effect of geometry conditioning on detection and direction for the three paired GCPR-Set models. Entries are recording-macro values averaged over seeds.

Measure	With Geometry	Without Geometry
*33,497 sampled frames; threshold 0.45*		
Precision at ten degrees	0.1280	0.0962
Recall at ten degrees	0.1589	0.1159
Mean predicted source count	1.411	1.293
*30,266 single-active-source frames; one output per frame*		
Mean angular error (°)	40.89	50.60
Mean error clipped at 30°	22.51	24.55
Hit rate at ten degrees	0.1385	0.0967

Precision and recall are averages of per-frame ratios. The single-output rows return the highest-existence slot of each model without a detection threshold.

**Table 10:** Gradient-norm measurements for GCPR-Set in 21 evaluations of development-validation batches at model checkpoints across three seeds. Entries are medians, with batch size sixteen.

Loss	Coefficient	Raw Norm (All)	Weighted (All)	Weighted (Shared)
Direction	1.00	3.2941	3.2941	2.4944
Existence	0.25	2.8410	0.7103	0.6659
Reconstruction	0.01	4.5820	0.0458	0.0444

Gradients are measured at selected checkpoints on validation batches with one or two active sources. Loss reductions and the weight schedule are given in Equations (7) and (8).

**Table 11:** Five-seed synthetic spherical comparison: 4000 matched scenes per model, with 800 per scenario. OSPA and full-minus-comparator contrasts are in degrees.

Scenario	Full	No Geom.	Fixed Order	Δ No Geom. [CI]	Δ Fixed [CI]
Aggregate	26.62	28.92	27.25	−2.29 [−2.51, −2.08]	−0.63 [−0.85, −0.40]
Nominal	26.75	28.86	27.63	−2.11 [−2.57, −1.68]	−0.88 [−1.35, −0.38]
Eight snapshots	25.72	28.96	27.16	−3.24 [−3.79, −2.70]	−1.44 [−1.71, −1.17]
5-mm position error	26.49	28.94	26.53	−2.45 [−3.00, −1.94]	−0.04 [−0.66, 0.63]
25% dropout	26.83	28.81	26.81	−1.98 [−2.47, −1.54]	0.02 [−0.50, 0.57]
Coherent sources	27.32	29.01	28.13	−1.69 [−2.13, −1.27]	−0.81 [−1.25, −0.36]

Models use synthetic pretraining without LOCATA fine-tuning. CIs resample matched generated batches; negative differences favor the full model. Sensor counts range from four to 32.

**Table 12:** Frequency-band stress on matched twelve-sensor, 0.30-m-aperture ULAs at −5 dB, using five pretraining seeds. OSPA and angular error are in degrees.

Band (Hz)	Frames	OSPA	Matched Angle	Δ vs. Training Band [CI]
500–3500	800	26.13	44.31	Reference
500–5500	800	28.70	52.11	2.57 [2.10, 3.07]
500–7000	800	29.06	58.12	2.94 [2.45, 3.43]
3500–7000	800	29.30	72.12	3.18 [2.71, 3.65]

Angular error is untruncated and conditional on matched sources. The 500–5500 Hz condition lies outside the training band but below the approximately 6.29-kHz nominal aliasing limit of this array.

**Table 13:** One-seed synthetic capacity pilot on 1280 paired scenes: three-query (K3) versus four-query (K4) set decoders. All true targets remain in evaluation.

True Count	Frames	K3 OSPA	K4 OSPA	Mean Count K3/K4	K4−K3 [CI]
0	137	13.80	30.00	1.07/3.54	16.20 [13.66, 19.18]
1	275	26.03	27.34	2.24/3.48	1.32 [0.54, 2.37]
2	291	26.91	25.68	2.42/3.86	−1.23 [−1.95, −0.48]
3	293	26.50	24.20	2.41/3.85	−2.30 [−3.14, −1.62]
4	284	27.28	23.84	2.43/3.84	−3.44 [−4.22, −2.54]

OSPA uses cutoff 30 degrees and threshold 0.45 and includes direction and cardinality errors.

**Table 14:** Joint-direction refinement for GCPR-Set on synthetic spherical scenes: one seed and twenty matched batches of eight scenes. OSPA and matched angular error are in degrees.

Method	OSPA	Matched Angle	Rec. Loss	CPU ms/Batch
No refinement	24.86	39.42	0.4520	0.00
Gradient (3 updates)	24.82	39.33	0.4509	18.26
Joint CG (up to 20)	24.63	38.88	0.4456	101.41

Both optimizers hold existence and powers fixed. CG receives up to twenty iterations versus three gradient updates. Times are refinement-only batch means and exclude the network forward pass.

**Table 15:** Batch-one median runtime of GCPR-Set in milliseconds on one measured frame per array. Network and classical-method timings use preloaded CSMs. Pipeline timing includes frontend processing of an already available audio window.

Array	*M*	Device	Model	Frontend	Pipeline	Coh-SRP	MUSIC
benchmark2	12	CPU	1.97	5.85	8.82	3.08	4.62
dicit	15	CPU	2.61	6.85	10.62	3.90	5.24
dummy	4	CPU	1.32	3.28	5.13	1.62	2.65
eigenmike	32	CPU	7.91	13.27	22.22	7.12	8.65
benchmark2	12	GPU	2.44	6.18	9.62	2.37	7.97
dicit	15	GPU	2.46	7.08	10.96	2.07	7.24
dummy	4	GPU	2.39	3.33	8.02	2.07	5.85
eigenmike	32	GPU	2.64	14.06	23.15	2.76	8.96

The GPU is an RTX 4060 Laptop, and one CPU thread is used. The frontend runs on CPU in both profiles. Pipeline timing excludes audio acquisition, stream scheduling, and cold-cache effects. Classical columns cover spectrum computation, counting, and peak selection from preloaded CSMs.

## Data Availability

LOCATA recordings and annotations are publicly available at https://doi.org/10.5281/zenodo.3630471. The original implementation is available at https://github.com/niubisile-wq/revision_tsp_20260807 (accessed on 29 October 2026), base commit 2efc5ca. Revision-specific materials, including the common-data query manifests, selection records, evaluation summaries, scoring scripts, and file checksums, accompany this article in the Supplementary Evidence archive. Additional predictions, model checkpoints, and training histories are available from the corresponding author on reasonable request. LOCATA audio is not redistributed.

## Supplementary Materials

The following supporting information can be downloaded at https://www.mdpi.com/article/10.3390/s26217004/s1, Supplementary Material, Sections S1–S4: model-version definitions, implementation and provenance checks, an evidence index, and the geometry readout analysis. The accompanying Supplementary Evidence archive includes query lists, selected configurations, numerical result tables, scoring scripts, compact original-model predictions for the geometry analysis, and a file-checksum manifest.

## Abbreviations

The following abbreviations are used in this manuscript:

AUC	Area under the receiver operating characteristic curve
BCE	Binary cross-entropy
CG	Conjugate gradient
CI	Confidence interval
Coh-SRP	Coherence-weighted steered-response power
CPU	Central processing unit
CSM	Cross-spectral matrix
CUDA	Compute Unified Device Architecture
DICIT	Distant talking Interfaces for Control of Interactive TV
DNN	Deep neural network
DOA	Direction of arrival
EM	Expectation–maximization
ESPRIT	Estimation of signal parameters via rotational invariance techniques
FP16	16-bit floating-point arithmetic
FP32	32-bit floating-point arithmetic
GCC	Generalized cross-correlation
GCPR	Geometry-conditioned pair representation
GCPR-DOA	Geometry-conditioned pair representation for DOA estimation
GCPR-Set	GCPR-DOA with the set decoder
GCPR-SM	GCPR-DOA with the spectral-moment decoder
GELU	Gaussian error linear unit
GPU	Graphics processing unit
JADED-RIP	Joint angle and delay estimator and detector via rotational invariance properties
LOCATA	Acoustic source LOCalization And TrAcking challenge
MEMS	Microelectromechanical systems
MLP	Multilayer perceptron
MUSIC	Multiple signal classification
NormMUSIC	Frequency-normalized MUSIC
OFDM	Orthogonal frequency-division multiplexing
OSPA	Optimal subpattern assignment
PSD	Positive-semidefinite
RT_60_	Reverberation time for a 60-dB energy decay
SBL	Sparse Bayesian learning
SD	Standard deviation
SNR	Signal-to-noise ratio
SRP	Steered-response power
SRP-PHAT	Steered-response power with phase transform
TDOA	Time-difference of arrival
ULA	Uniform linear array
