# Supplementary material for: Geometry-Conditioned Pair Representation and Covariance Reconstruction for Multi-Source Direction-of-Arrival Estimation with Microphone Arrays
Source: Sensors (Basel). 2026 Nov 5;26(21):7004. doi: 10.3390/s26217004 (PMC13766416; doi:10.3390/s26217004)
Supplement: Supplementary file 1 [file sensors-26-07004-s001.zip › Supplementary_Material.pdf]

Supplementary Material

Geometry-Conditioned Pair Representation and Covariance Reconstruction for Multi-Source Direction-of-Arrival Estimation with Microphone Arrays

Sensors-4527743

26 October 2026

S1 Model and training configurations

The *original GCPR model* was used in the experiments reported in the submitted manuscript. The additional comparison uses a *common-data GCPR configuration* that retains the geometry-conditioned pair-to-set design but uses a separate implementation and different training settings. The table below maps these model names to the identifiers in the evidence files and the corresponding experiments.

Table S1: Model configurations and evaluated comparisons.

	Original GCPR model	Common-data GCPR configuration
Stored model label	Archived `full` and paired `no_geometry/no_reconstruction` rows	`gcpr_physics_candidate`; SRP-DNN comparator: `srp_dnn_query_adaptation`
Training	Thirty synthetic-pretraining epochs followed by five benchmark2 fine-tuning epochs	From scratch, 21,383 common training queries, 20 epochs, four array layouts
Initialization	Explicit unit-power initialization in the archived protocol	Constructor initialization of power heads
Primary seeds	Paired components: 20260807–20260809	Each learned method: 2026101402 and 2026101404
Evaluation sample	Archived one-/two-active-source primary sample: 33,497 frames	46,457 queries, including silence; common single-source diagnostic: 15,185 queries
Comparison	Within-architecture component contrasts under paired settings	Fixed-budget comparison with common query exposure and development selection

The additional comparison supplies both methods with the same waveforms, queries, annotations, sensors and geometry. SRP-DNN is adapted to this query protocol through temporal interpolation, query-level pairwise supervision and pair microbatches of at most sixteen pairs for BatchNorm. Acoustic representations and objectives remain method-specific. The experiment compares the specified implementations using the same data and update budget; convergence, equal computational cost and performance after method-specific hyperparameter optimization were not assessed.

S2 Implementation checks and provenance

S2.1 Training records, selection and package coverage

The frozen common-data settings specify AdamW with a learning rate 3×10^{-4}, weight decay 10^{-4}, gradient clipping at 1, one query per microbatch and accumulation over four queries. Training and development contain 21,383 and 6,144 queries. Selection considers checkpoints at epochs 5, 10, 15 and 20 and thresholds 0.05–0.95 in increments of 0.05, using development group-macro OSPA with the documented tie-breaking rules based on F1, threshold and epoch. The packaged manifests preserve query identities and label digests.

The retained completion receipt reports four learned runs, sixteen development validations and the final six-case aggregate. Each of the four packaged selection records identifies the selected epoch, threshold and checkpoint SHA-256 digest. The hashes of the final aggregate and all six summary files match those in the exported evidence index.

The historical protocol narrative reports an independent audit of twenty consecutive epochs, 21,383 queries processed per epoch and a digest for each epoch checkpoint in every learned run. This compact package contains the final completion receipt, selected-checkpoint records and result summaries. Repeating the full eighty-checkpoint verification requires the complete final four-run epoch audit and consumed-query logs, which were not located in the available files. Earlier incomplete training snapshots are excluded.

S2.2 Original-model files and result aggregation

The identity report links the submitted manuscript and result-table sources to the recovered archive by file hashes, verifies 96 source files, and independently reaggregates the main tables. Of its 3,305 checksum entries, 3,011 present entries were verified and 294 input-cache index files were missing; every present file matched its recorded hash. These checks cover model/result provenance and table aggregation. The archive remains incomplete, and original retraining and fresh waveform-to-prediction inference are outside these checks. The component-reaggregation report reproduces the three-seed geometry and reconstruction contrasts.

S2.3 Covariance-convention correspondence

For an array-to-source direction u, the common-data configuration uses

$$R_{f,mn} = \mathbb{E}[X_{f,m}\overline{X_{f,n}}], \qquad a_m(u,f) = \exp(+j2\pi f p_m^{\mathsf{T}} u/c).$$

The covariance obtained under the archived conjugated-observation convention is the complex conjugate of this covariance when the conventions are applied consistently. For the full-feature network branches, the explicit mapping conjugates the input CSM and negates columns 1 and 4 (zero-based) of the first pair-encoder weight matrix, corresponding to the imaginary normalized-CSM and coherence features; other parameters are retained.

The check uses one saved manuscript example and nine synthetic batches spanning 4, 8 or 12 sensors; 1, 4 or 8 frequency bins; valid-sensor masks; and zero to three targets. Forward outputs, matched-weight loss components and gradients after the same mapping agree within absolute and relative tolerances of 10^{-5} in all ten cases. This verifies arithmetic correspondence at mapped weights in the full-feature mode. Constructor initialization, optimization trajectories and learned accuracy are outside this arithmetic comparison. The submitted and current learned weights are unchanged.

The retained phase-v2 source already applies the measured-frontend convention and frequency averaging of the scalar off-diagonal reconstruction loss. In the common-data configuration, direction and existence loss weights are 1 and 0.25, and the reconstruction coefficient is $0.01\min(1, e/5)$ for zero-based epoch e. The archived component contrasts were measured under the original training settings; their effects under the common-data settings require a corresponding ablation.

S3 Reproduced tables and material index

S3.1 Directly inspectable numerical evidence

Table S2 gives means recomputed from the retained paired-seed rows. The source paired CSV contains intervals from physical-recording-cluster resampling with the trained pairs held fixed. They quantify recording-level uncertainty conditional on those models. All differences are computed as *full minus ablated*, so lower OSPA and higher F1 favor the full model.

Table S2: Original-model component contrasts, seeds 20260807–20260809.

Comparison	OSPA (°)	Localized F1@10	Exact-count accuracy (pp)
Full minus no geometry	−1.3492	+0.03436	−7.6102
Full minus no reconstruction	−0.7808	+0.00405	−4.7464

Table S3: Common-data results. Seeds are abbreviated by their final four digits.

Method / seed	Macro OSPA (°)	Active F1@10	Single-source median (°)
Common-data GCPR / 1402	18.760	0.000542	26.63
Common-data GCPR / 1404	18.764	0.000215	26.73
SRP-DNN query adaptation / 1402	11.926	0.468389	9.02
SRP-DNN query adaptation / 1404	11.426	0.477086	8.77
Covariance SBL (EM)	18.888	0.050123	17.07
Archived SBL counting control	26.617	0.231389	13.94

For the results in Table S3, OSPA uses order 1, a 30° cutoff and equal weighting across groups defined by array, task and true source count, including silent groups. Active F1 excludes silent queries. The single-source diagnostic returns one direction without thresholding; reference labels define the cohort, while the model readout selects the direction. GCPR produces empty sets on 35,600 and 35,610 of 35,642 active queries, combining a low silent-query false-detection rate with severe active-source misses. The six stored summaries and case hashes permit inspection of the reported scores. Recomputing them requires the raw common-data predictions, which are excluded from this compact package.

S3.2 Evidence package and reproduction instructions

`Supplementary_Evidence.zip` accompanies this supplement. `README.txt` gives instructions and input requirements; `INDEX.csv` maps each item to its project-relative source and hash. `SHA256SUMS.txt` covers every delivered evidence file. Running `python reproduce_tables.py` verifies the package, reproduces sixteen archived paired-table means and checks the common-data summary hashes. The generated receipt records these table and file checks.

Package directory	Supplied material
`tables/`	Original per-seed and paired CSVs; common-data table CSV, evidence index and descriptive statistics.
`manifests/`	Frozen training, development and evaluation query identities and label digests.
`configs/`	Scientific-setting extract with the original frozen-protocol hash and an explicit transformation note.
`checks/`	Original identity and component checks, covariance mapping report, completion receipt, four selection records and six final summaries.
`code/`	Existing original and common-data scoring/selection/aggregation source files; dependency and input limitations are stated in the README.
`geometry_readout/`	Six compressed original-model prediction tables and independent replay of the post hoc geometry readout in Section S4.

This release provides the table reproduction and stored-result checks described above. It excludes LOCATA audio, large checkpoint weights, full training-consumption logs and raw common-data predictions. The 294 historical cache-index files remain unavailable. LOCATA data must be obtained separately under its access terms; the supplied files alone are insufficient for full training and waveform inference. The current training, development and evaluation cohorts use disjoint physical recordings. LOCATA evaluation results had already been inspected during the original study.

S4 Original-Model Geometry Readout Analysis

This post hoc analysis uses the original full-model and no-geometry predictions from runs with seeds 20260807–20260809, with the trained models, thresholds and checkpoints unchanged. These are the original-model predictions, separate from the common-data experiment. The six prediction-file SHA-256 digests match their completion records. Reaggregating their 33,497 primary frames with the original sampling weights reproduces the archived OSPA, count accuracy and localized F1 tables.

Precision at ten degrees is computed per frame as $\mathrm{TP}_{10}/\max(\widehat{K}, 1)$ and recall as TP_{10}/K. The original threshold 0.45 and zero-refinement setting are retained. These per-frame ratios are averaged using sampling-bin weights within each array-recording and then equally across the 132 array-recordings.

The fixed single-output readout returns the slot with the highest existence probability, with the lowest slot index used to break ties. Output selection uses these probabilities without a detection threshold; reference labels define the single-active-source cohort. All six runs use the same 30,266 primary frames, including single-active intervals in multiple-source tasks. The weighted sample represents 297,170 eligible frames. We retain the within-recording sampling weights and equal-recording aggregation on this subset.

Table S4: Three-seed means for the original models. The first three metrics use all 33,497 primary frames; the final two use the common 30,266-frame single-active-source subset with one returned direction.

Model	Precision@10	Recall@10	Mean $\widehat{K}$	Angle (°)	Within 10°
Full	0.127989	0.158890	1.411398	40.887858	0.138505
No geometry	0.096207	0.115914	1.293124	50.596574	0.096666

The full-minus-no-geometry difference in mean untruncated angular error under the single-output readout is $-9.709°$, with a conditional 95% physical-recording-cluster interval of $[-14.072, -5.920]°$. The seed differences are -10.407, -3.046, and $-15.674°$. The corresponding thirty-degree-clipped means are 22.507 and 24.551 degrees. The ten-degree hit-rate difference is 0.041839, with an interval of $[0.018905, 0.064441]$. Intervals use 10,000 resamples of the 56 physical-recording clusters and keep simultaneous array recordings together. The three trained pairs remain fixed throughout resampling.

Geometry also reduces angular error when both models return one direction. This readout measures the joint effect of probability ranking and direction prediction. Absolute angular errors remain large. The analysis reuses the original, previously inspected evaluation predictions.

The six compressed primary-frame CSVs retain all slot directions, existence probabilities, target directions, weights, and original scalar scores. `replay_compact_geometry.py` recomputes the point estimates and paired intervals without audio or model weights. Independent float64 matching reproduced every archived TP_{10} exactly and the new table values within an absolute difference of 1.5×10^{-14}; the largest difference from the original FP32 OSPA values was 0.0117°. Per-seed values, secondary Task 1/3/5 results, source hashes, and bootstrap intervals accompany this analysis.
