# Supplementary material for: Geometry-Conditioned Pair Representation and Covariance Reconstruction for Multi-Source Direction-of-Arrival Estimation with Microphone Arrays
Source: Sensors (Basel). 2026 Nov 5;26(21):7004. doi: 10.3390/s26217004 (PMC13766416; doi:10.3390/s26217004)
Supplement: Supplementary file 1 [file sensors-26-07004-s001.zip › Supplementary_Evidence/Supplementary_Evidence/docs/Supplementary_Material.pdf]

Supplementary Material

Geometry-Conditioned Pair Representation and Covariance Reconstruction for Multi-Source Direction-of-Arrival Estimation with Microphone Arrays

Sensors-4527743

26 October 2026

S1 Protocol identity and scope

This supplement distinguishes the *original GCPR model* used in the submitted experiments from the *common-data GCPR configuration* used in the additional comparison. The latter retains a geometry-conditioned pair-to-set construction but uses a separate implementation and training configuration. The model-version table in the main text gives the corresponding scope; the identities below also map the readable names to the unchanged identifiers in the evidence files.

Table S1: Version identities and the comparisons each supports.

	Original GCPR model	Common-data GCPR configuration
Evidence identity	Archived `full` and paired `no_geometry/no_reconstruction` rows	`gcpr_physics_candidate`; SRP-DNN comparator: `srp_dnn_query_adaptation`
Training	Thirty synthetic-pretraining epochs followed by five benchmark2 fine-tuning epochs	From scratch, 21,383 common training queries, 20 epochs, four array layouts
Initialization	Explicit unit-power initialization in the archived protocol	Constructor initialization of power heads
Primary seeds	Paired components: 20260807–20260809	Each learned method: 2026101402 and 2026101404
Evaluation identity	Archived one-/two-active-source primary sample: 33,497 frames	46,457 queries, including silence; common single-source diagnostic: 15,185 queries
Supported inference	Within-architecture component contrasts under paired settings	Fixed-budget comparison of two stated implementations with common query exposure and development selection

The two cohorts and training versions are not interchangeable. Shared waveforms, queries, annotations, sensors and geometry control data exposure in the additional comparison. Acoustic representations and objectives remain method-specific; SRP-DNN uses temporal interpolation, query-level pair supervision and pair microbatches of at most sixteen pairs for BatchNorm. An adapted baseline can support this stated comparison without being an exact native-protocol reproduction. Matching epochs and update schedules does not establish convergence, equal computation or method-optimal hyperparameter tuning.

S2 Implementation checks and provenance

S2.1 Training records, selection and package coverage

The frozen common-data settings specify AdamW, learning rate 3×10^{-4}, weight decay 10^{-4}, gradient clipping at 1, one query per microbatch and accumulation over four queries. Training and development contain 21,383 and 6,144 queries. Selection considers epochs 5, 10, 15 and 20 and thresholds 0.05–0.95 at increments of 0.05, using development group-macro OSPA with the documented F1, threshold and epoch tie rules. The packaged manifests preserve query identities and label digests.

The retained imported completion receipt reports four learned runs, sixteen development validations and the final six-case aggregate. Each of the four packaged selection records identifies its selected epoch, threshold

and checkpoint SHA-256 digest. The final aggregate and all six summary files were checked against the hashes in the exported evidence index during preparation of this package.

The historical protocol narrative reports an independent audit of twenty consecutive epochs, 21,383 consumed queries per epoch and an epoch-checkpoint digest for each learned run. The complete final four-run epoch audit and consumed-query logs were not located among the locally available files used for this compact package. Consequently, the supplied receipt and selected-checkpoint records do not let a reader repeat that full eighty-checkpoint verification. Earlier incomplete training snapshots are not used as substitutes. This is a package-coverage limitation, distinct from the retained final result summaries.

S2.2 Original-model identity and archived aggregation

The retained identity report links the submitted manuscript and result-table sources to the recovered archive by file hashes, verifies 96 source files, and independently reaggregates the archived main tables. In its 3,305-entry scientific checksum inventory, 3,011 present entries were verified and 294 input-cache index files were missing; no present-file mismatch was recorded. Thus, model/result provenance and table aggregation are supported, while complete archive recovery, original retraining and fresh waveform-to-prediction reproduction are not claimed. The packaged component-reaggregation report reproduces the three-seed geometry and reconstruction contrasts and preserves their scope.

S2.3 Covariance-convention correspondence

For array-to-source direction u, the common-data configuration uses

$$R_{f,mn} = \mathbb{E}[X_{f,m}\overline{X_{f,n}}], \qquad a_m(u,f) = \exp(+j2\pi f p_m^{\top} u/c).$$

The archived conjugated-observation convention is its complex conjugate when applied consistently. For the full-feature network branches, the explicit mapping conjugates the input CSM and negates columns 1 and 4 (zero-based) of the first pair-encoder weight matrix, corresponding to the imaginary normalized-CSM and coherence features; other parameters are retained.

The recorded check comprises one saved manuscript example and nine synthetic batches spanning 4, 8 or 12 sensors; 1, 4 or 8 frequency bins; valid-sensor masks; and zero to three targets. Forward outputs, matched-weight loss components and gradients after the same mapping satisfy absolute and relative tolerances of 10^{-5} in all ten cases. This checks arithmetic correspondence in the full-feature mode. It does not establish equal constructor initialization, optimization trajectories or learned accuracy, and the submitted or current learned weights were not modified by the check.

The retained phase-v2 source already applies the measured-frontend convention and frequency averaging of the scalar off-diagonal reconstruction loss. Neither is presented as a newly demonstrated repair to the submitted model. In the common-data configuration, direction and existence weights are 1 and 0.25, and the reconstruction coefficient is $0.01\min(1, e/5)$ for zero-based epoch e. Effects measured by archived component ablations cannot simply be transferred to this training configuration.

S3 Reproduced tables and material index

S3.1 Directly inspectable numerical evidence

Table S2 shows means recomputed from the retained paired-seed rows. The intervals in the source paired CSV condition on the trained model pairs and resample physical-recording clusters; they do not represent uncertainty over all possible training runs. All signs below are *full minus ablated*, so lower OSPA and higher F1 favor the full model.

Table S2: Original-model component contrasts, seeds 20260807–20260809.

Comparison	OSPA (°)	Localized F1@10	Exact-count accuracy (pp)
Full minus no geometry	−1.3492	+0.03436	−7.6102
Full minus no reconstruction	−0.7808	+0.00405	−4.7464

Table S3: Common-data results. Seeds are abbreviated by their final four digits.

Method / seed	Macro OSPA (°)	Active F1@10	Single-source median (°)
Common-data GCPR / 1402	18.760	0.000542	26.63
Common-data GCPR / 1404	18.764	0.000215	26.73
SRP-DNN query adaptation / 1402	11.926	0.468389	9.02
SRP-DNN query adaptation / 1404	11.426	0.477086	8.77
Covariance SBL (EM)	18.888	0.050123	17.07
Archived SBL counting control	26.617	0.231389	13.94

For the common-data results in Table S3, OSPA uses order 1, a 30° cutoff and equal array/task/true-source-count group weighting, including silence. Active F1 excludes silent queries. The single-source diagnostic uses one direction per query without a detection threshold; the reference selects the cohort, not an output by proximity. The GCPR configuration produces empty sets on 35,600 and 35,610 of 35,642 active queries. Its low silent-query false-detection rate is therefore not a useful detection advantage. The six stored summaries and case hashes permit inspection of these reported results; recomputation from omitted raw common-data predictions requires those predictions separately.

S3.2 Evidence package and reproduction boundary

`Supplementary_Evidence.zip` accompanies this supplement. `README.txt` explains use and limitations; `INDEX.csv` maps each copied or extracted item to its project-relative source and hash. `SHA256SUMS.txt` covers every delivered evidence file. Running `python reproduce_tables.py` verifies the package, reproduces sixteen archived paired-table means and checks the common-data summary hashes; the generated receipt explicitly distinguishes these checks from waveform inference or training.

Package directory	Supplied material
`tables/`	Original per-seed and paired CSVs; common-data table CSV, evidence index and descriptive statistics.
`manifests/`	Frozen training, development and evaluation query identities and label digests.
`configs/`	Scientific-setting extract with the original frozen-protocol hash and an explicit transformation note.
`checks/`	Original identity and component checks, covariance mapping report, completion receipt, four selection records and six final summaries.
`code/`	Existing original and common-data scoring/selection/aggregation source files; dependency and input limitations are stated in the README.
`geometry_readout/`	Six compressed original-model prediction tables and independent replay of the post hoc geometry readout in Section S4.

This compact release excludes LOCATA audio, large checkpoint weights, full training-consumption logs and raw common-data predictions. The 294 missing historical cache-index files remain unavailable. No public repository or permanent access URL is claimed. The package supplies inspectable evidence and limited table reproduction, not a complete executable training distribution. LOCATA data must be obtained separately under its access terms. Earlier evaluation inspection is disclosed; recording-disjoint cohorts do not provide a new blind confirmation.

S4 Original-Model Geometry Readout Analysis

This post hoc analysis uses the retained original full-model and no-geometry predictions at seeds 20260807–20260809. It does not use the common-data GCPR configuration or change any trained model, threshold, or checkpoint. The six original prediction-file SHA-256 digests match their completion records. Their 33,497 primary frames, sampling weights, and original OSPA, count accuracy, and localized F1 scores reproduce the archived tables.

Precision at ten degrees is computed per frame as $\mathrm{TP}_{10}/\max(\widehat{K},1)$ and recall as TP_{10}/K. The original threshold 0.45 and zero-refinement setting are retained. Per-frame values are averaged using sampling-bin weights within each array-recording and then equally across the 132 array-recordings. These are means of per-frame precision and recall, not ratios of pooled detection totals.

To assess whether the gain depends entirely on returning more directions, a second readout always returns exactly one direction: the slot with the highest existence probability, with the first index resolving ties. No detection threshold or reference-based slot selection is used. All six runs use the same 30,266 primary frames with exactly one active reference, including such intervals in multiple-source tasks. Their weights represent 297,170 eligible frames. We retain the within-recording sampling weights and equal-recording aggregation on this subset.

Table S4: Three-seed means for the original models. The first three metrics use all 33,497 primary frames; the final two use the common 30,266-frame single-active-source subset with one returned direction.

Model	Precision@10	Recall@10	Mean $\widehat{K}$	Angle (°)	Within 10°
Full	0.127989	0.158890	1.411398	40.887858	0.138505
No geometry	0.096207	0.115914	1.293124	50.596574	0.096666

The full-minus-no-geometry difference in untruncated single-direction mean error is $-9.709°$, with a conditional 95% physical-recording-cluster interval of $[-14.072, -5.920]°$. The seed differences are -10.407, -3.046, and $-15.674°$. The corresponding thirty-degree-clipped means are 22.507 and 24.551 degrees. The ten-degree hit-rate difference is 0.041839, with interval $[0.018905, 0.064441]$. Intervals use 10,000 resamples of the 56 physical-recording clusters, preserve simultaneous arrays, and condition on the three trained pairs; they do not quantify general training-seed uncertainty.

These results show that the geometry benefit is not explained solely by the larger predicted sets at the original threshold. The single-direction readout still combines probability ranking with direction prediction, so it does not isolate their individual mechanisms. Absolute angular errors remain large. The analysis reuses previously inspected evaluation predictions and is not an independent confirmation or a performance repair.

The six compressed primary-frame CSVs retain all slot directions, existence probabilities, target directions, weights, and original scalar scores. `replay_compact_geometry.py` recomputes the point estimates and paired intervals without audio or model weights. Independent float64 matching reproduced every archived TP_{10} exactly and the new table values to 1.5×10^{-14}; the largest difference from original FP32 OSPA was 0.0117°. Per-seed values, secondary Task 1/3/5 results, source hashes, and bootstrap intervals accompany this analysis.
